# Tethered agonist– and GAIN domain–independent signaling of an adhesion GPCR

**DOI:** 10.1126/sciadv.adu3822

**Published:** 2026-07-01

**Authors:** Jie Wang, Yi Miao, Jinzhao Wang, Shaoyuan Zhu, Yu Zhang, Tsz Lok Wong, Ahmed Yousif, Marius Wernig, Thomas C. Südhof

**Affiliations:** ^1^ Department of Molecular and Cellular Physiology, Stanford University School of Medicine, Stanford, CA 94305, USA.; ^2^ Howard Hughes Medical Institute, Stanford University School of Medicine, Stanford, CA 94305, USA.; ^3^ Division of Life Science, SIAT-HKUST Joint Laboratory for Brain Science, The Hong Kong University of Science and Technology, Hong Kong, China.; ^4^ Institute for Stem Cell Biology and Regenerative Medicine, Department of Pathology, Stanford University School of Medicine, Stanford, CA 94305, USA.

## Abstract

In transfected cells, adhesion G protein–coupled receptors (GPCRs) are activated by tethered agonists that are embedded in their canonical autoproteolytic GAIN domain. It is unknown, however, whether a tethered agonist–dependent activation mechanism generally mediates the physiological functions of adhesion GPCRs. Here, we show that G protein signaling by BAI3 (*Adgrb3*), a brain-specific adhesion GPCR, is essential for its functions in controlling axon and dendrite growth and promoting synapse formation. Moreover, our signal transduction assays confirm that constitutive exposure of BAI3’s tethered agonist massively stimulates (~5-fold) its GPCR activity. However, the constitutive exposure of BAI3’s tethered agonist, produced by deletion of its extracellular domains, blocked instead of activating BAI3’s functions in regulating axonal and dendritic growth and promoting synapse formation. Moreover, inactivating mutations of BAI3’s tethered agonist or deletion of BAI3’s constituent GAIN domain did not detectably impair BAI3’s physiological functions. Thus, the GPCR activity of BAI3 is functionally required, whereas tethered agonist–mediated stimulation of its GPCR activity is not.

## Teaser

The GPCR activity of BAI3 is functionally required, whereas tethered agonist–mediated stimulation of GPCR signaling is not.

## INTRODUCTION

Adhesion G protein–coupled receptors (GPCRs), the second largest GPCR subfamily, play diverse functions in human physiology and disease (*1*–*3*). Most adhesion GPCRs comprise multiple extracellular domains (ECDs) that include a canonical GAIN domain (for “GPCR autoproteolysis inducing”) (*4*), a classical seven-transmembrane GPCR moiety, and a large cytoplasmic sequence (*5*). The GAIN domain undergoes autoproteolysis, thereby cleaving adhesion GPCRs into two noncovalently linked fragments: an N-terminal fragment containing the ECDs and a C-terminal fragment that includes the GPCR moiety and cytoplasmic region (*4*, *6*). Although autoproteolysis does not dissociate the N- and C-terminal fragments, adhesion forces can pull the N-terminal fragment containing the ECDs away from the GPCR moiety (*5*). When this occurs, the short extracellular peptide that forms the N terminus of the C-terminal fragment is exposed, which then inserts into the ligand-binding pocket of the GPCR moiety and activates adhesion GPCR signaling as a tethered agonist (*7*–*12*). Consistent with the tethered agonist mechanism of adhesion GPCR activation, cryo–electron microscopy structures revealed that the tethered agonist is inserted in the ligand-binding pocket of the GPCR moiety of adhesion GPCRs when the ECDs are deleted (*7*–*9*, *13*–*15*). Some adhesion GPCRs may not be cleaved at the GAIN domain, in which case it is envisioned that the tethered agonist is exposed by partial unfolding of the GAIN domain and thereby activates the GPCR (*16*). Thus, the now dominant model for the function of adhesion GPCRs proposes that the mechanical dissociation of adhesion GPCR ECDs or partial GAIN domain unfolding exposes a tethered agonist, which then activates the GPCR (*7*–*9*, *11*). However, many of the experiments supporting this model were performed in transfected cancer cells. The physiological functions of tethered agonists remain untested for most adhesion GPCRs, and, for many adhesion GPCRs, it is even unclear whether they function as GPCRs at all.

**Fig. 1.**
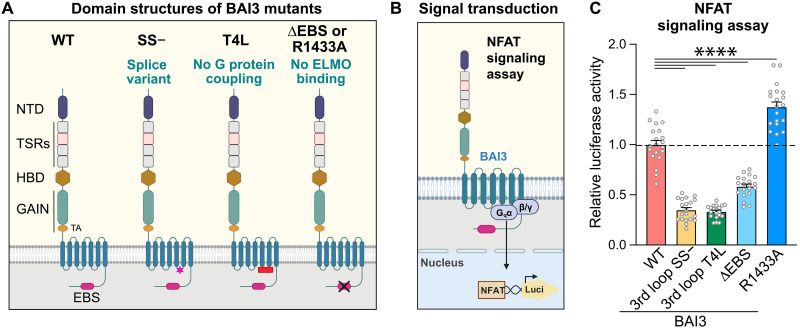
Domain structures and NFAT signaling activity of WT and mutant BAI3 proteins. (**A**) Domain structures of wild-type (WT) and mutant BAI3 proteins. The ECDs of BAI3 comprise an N-terminal Cub-like domain (NTD), four thrombospondin repeats (TSRs), a “hormone-binding domain” (HBD), and a canonical GAIN domain that includes the tethered agonist (TA). Following the ECDs, BAI3 contains a classical GPCR moiety with seven transmembrane regions and an extensive cytoplasmic tail that includes an ELMO-binding sequence (EBS). The four BAI3 mutants analyzed in Figs. 1 to 4 feature a rare alternatively spliced BAI3 variant in the third cytoplasmic loop (SS−), an insertion of T4 lysozyme into the third cytoplasmic loop to disrupt G protein coupling (“T4L”), a deletion of the EBS (DEBS), and a point mutation in the EBS that disrupts ELMO binding (R1433A) based on the atomic structure of the BAI3 EBS in a complex with ELMO (*29*). Created in BioRender. Y. Miao (2026), https://BioRender.com/f6kp3kp. (**B**) Schematic of the nuclear factor of activated T cell (NFAT) signaling assay used to assess the effect of BAI3 mutants on GPCR signaling in HEK293T cells. BAI3-induced G_q_ activation stimulates NFAT that, in turn, drives transcription of a luciferase reporter. Created in BioRender. Y. Miao (2026), https://BioRender.com/edk6p4d. (**C**) WT BAI3 exhibits robust constitutive NFAT signaling activity in transfected HEK293T cells that is impaired by the SS− splice variant, the T4L insertion in the third cytoplasmic loop, and the EBS deletion but not by the EBS R1433A point mutation. *N* (samples/experiments) = 20/4. Data are means ± SEM. Statistical significance was determined using one-way analysis of variance (ANOVA) followed by Dunnett’s multiple comparisons test comparing all conditions to the WT controls (****P < 0.0001).

Here, we aimed to explore the biological role of the tethered agonist–mediated stimulation of adhesion GPCRs by focusing on BAI3 (gene symbol *Adgrb3*). Despite being named “brain angiogenesis inhibitor 3,” BAI3 is not primarily involved in angiogenesis but regulates neuronal development (*17*–*19*). We selected BAI3 instead of BAI1 or BAI2 for the current project because BAI3 performs multiple functions that can be readily analyzed in cultured neurons (*20*). BAI3 contains a classical GAIN domain that does not undergo autoproteolysis when overexpressed in human embryonic kidney (HEK) 293 cells, although endogenous BAI3 in mouse brain may be cleaved (*4*). In addition to the GAIN domain, the N-terminal ECDs of BAI3 comprise a noncanonical CUB-like domain, four thrombospondin type 1 repeats (TSRs), and a hormone-binding domain (HBD) (Fig. 1A). The N-terminal CUB-like domain of BAI3 binds to C1qls, a family of secreted C1q-domain proteins (*21*–*24*), whereas the second TSR binds to RTN4R’s, a family of glycosylphosphatidylinositol-anchored leucine-rich repeat proteins (*25*). Following the GAIN domain, BAI3 includes a classical GPCR moiety and a long cytoplasmic sequence that includes an ELMO-binding sequence (EBS), implicating BAI3 in Rho/Rac signaling (*26*–*29*). Additionally, the C terminus of BAI3 contains a PDZ-binding motif, indicating a potential interaction with PDZ domain–containing proteins (*30*–*32*).

BAI3 plays important roles in neuronal development by promoting synapse formation (*20*, *22*, *24*, *33*–*36*) and restricting the growth of dendrites (*20*, *25*, *28*) and axons (*20*, *25*). Similar, although sometimes divergent, results were obtained for BAI1 (*20*, *37*–*39*) and BAI2 (*40*). The molecular mechanisms underlying these physiological functions of BAI3 and BAI1 are incompletely understood. C1ql binding to BAI3 is crucial for synapse formation in the cerebellum and olfactory bulb (*22*, *33*–*35*), while the binding site for RTN4R’s is required for all BAI3 functions tested (*20*). Furthermore, ELMO binding to BAI3 mediates the control of dendritic arborization (*28*) but is dispensable for BAI3’s role in promoting climbing-fiber synapse formation (*35*). These studies uncovered a rich biology of BAI3 functions. However, these studies did not reveal whether (i) the GPCR activity of BAI3 mediates any of its functions, (ii) the regulation of the GPCR activity by a tethered agonist controls BAI3’s roles in neuronal development, (iii) the EBS is essential for all of BAI3’s functions, and (iv) BAI3’s functions in axon and dendrite growth and in synapse formation involve similar mechanisms.

Here, we have addressed these knowledge gaps using rescue experiments in primary neuron-glia cultures obtained from newborn conditional BAI3 knockout mice (*22*). Our results reveal that BAI3 acts as a true GPCR in its diverse functions but that its tethered agonist is dispensable for all of the three BAI3 functions that we analyzed. Moreover, we show that the EBS of BAI3 is essential for its role in controlling axonal and dendritic growth, but not for synapse formation, mechanistically dissociating these activities of BAI3. Critically, deletions of the entire GAIN domain and HBD of BAI3 did not majorly impair its function in axonal and dendritic arborizations or synaptic transmission, suggesting that, despite the universal presence of a GAIN domain in adhesion GPCRs, the GAIN domain is not actually required for at least a subset of adhesion GPCR functions.

## RESULTS

### BAI3 GPCR signaling is required for BAI3-mediated control of axonal and dendritic arborizations and synapse formation

We first asked whether the GPCR activity of BAI3 is functionally required and whether ELMO binding to BAI3’s cytoplasmic sequences via its EBS is involved. For this purpose, we generated two BAI3 variants designed to impair GPCR signaling: an insertion of T4 lysozyme into the third cytoplasmic loop of the GPCR moiety (T4L) (*41*) and a short deletion of a sequence in the third cytoplasmic loop that corresponds to a naturally occurring alternatively spliced variant of BAI3 (SS−) (Fig. 1A) (*42*). In addition, we constructed two mutants that disrupt ELMO binding: a deletion of the entire EBS (ΔEBS) and a point mutation that, based on the crystal structure of the BAI1-ELMO complex, abolishes ELMO binding (R1433A) (Fig. 1A) (*29*). Using a transcriptional GPCR activity assay in HEK293T cells with nuclear factor of activated T cell (NFAT) activation as a readout (Fig. 1B) (*43*), we confirmed that BAI3 exhibits robust constitutive GPCR activity (Fig. 1C). This activity was suppressed by the T4L and SS− mutations, modestly decreased by the ΔEBS mutation, but modestly increased by the R1433A mutation (Fig. 1C). These results validate the utility of the mutations designed to disrupt the GPCR activity. We do not quite understand why the ΔEBS mutation also affected the BAI3 GPCR activity, especially because the R1433A did not, suggesting a possible nonspecific effect or a cross-talk of the EBS with G protein signaling.

**Fig. 2.**
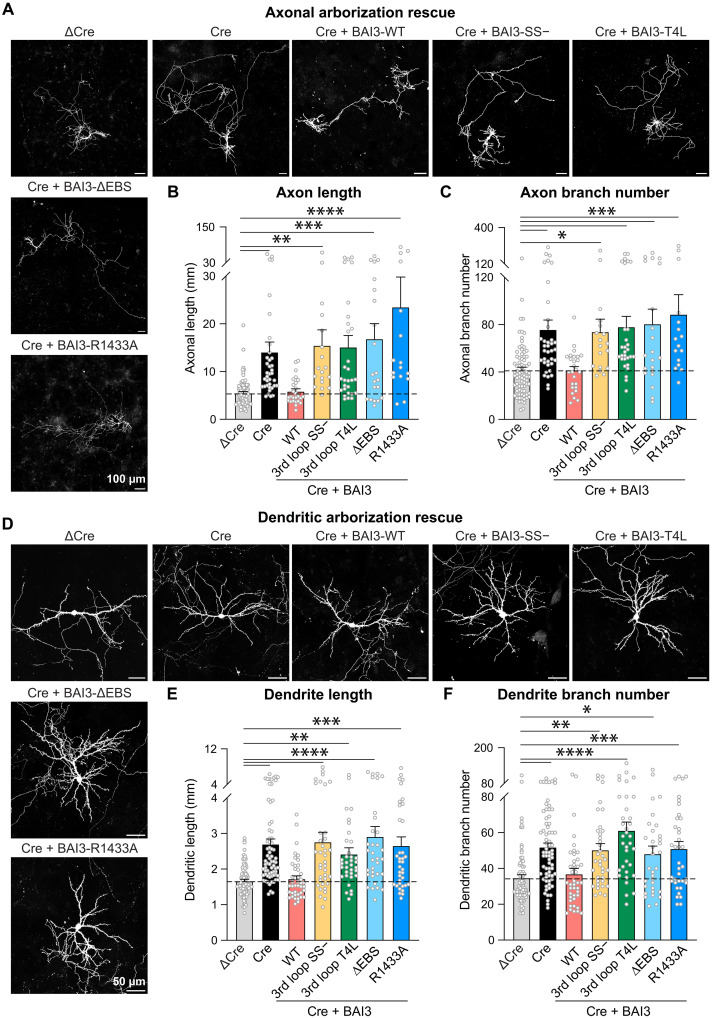
BAI3’s GPCR activity and ELMO binding are essential for BAI3’s function in restricting axonal and dendritic arborizations in hippocampal neurons. (**A**) Representative images of cultured hippocampal neurons from experiments that quantify the effect of BAI3 mutations on axon length and axon branching. In these and subsequent experiments, mixed neuron-glia cultures from newborn BAI3 conditional KO mice were infected at DIV4 with lentiviruses expressing ΔCre–enhanced green fluorescent protein (EGFP) (control) or Cre-EGFP and at DIV6 with lentiviruses expressing WT or mutant BAI3s. For analysis of axons and dendrites, neurons were sparsely transfected at DIV9 with tdTomato plasmids to label individual neurons and analyzed at DIV14 (fig. S1A). (**B** and **C**) Summary graphs of the length (B) and branch numbers (C) of axons as a function of the expression of WT or mutant BAI3 in mixed neuron-glia cultures. *N* (cells/experiments) = 79/20, 37/14, 26/8, 18/8, 29/10, 21/7, and 16/9 from left to right. (**D**) Representative images illustrating dendrite length and branching in hippocampal neurons from mixed neuron-glia cultures of BAI3 cKO mice. The experiments were performed as described for (A), except that dendrites in the images were analyzed at higher magnification. (**E** and **F**) Summary graphs of the length (E) and branch numbers (F) of dendrites as a function of the expression of WT or mutant BAI3. The four mutants that block the restriction of axonal arborization by BAI3 also impair the control of dendritic arborization by BAI3. *N* (cells/experiments) = 79/11, 70/11, 42/7, 37/6, 35/4, 33/7, and 37/6 from left to right. Data in (B), (C), (E), and (F) are means ± SEM. Statistical significance was determined using one-way ANOVA followed by Dunnett’s multiple comparisons test comparing all conditions to ΔCre controls (*P < 0.05, **P < 0.01, ***P < 0.001, and ****P < 0.0001).

Next, we tested the competence of wild-type (WT) and mutant BAI3 to rescue the disinhibiting effect of the BAI3 deletion on axonal and dendritic growth (Fig. 2, A to F; and fig. S2, A to D). We prepared mixed neuron-glia hippocampal cultures from newborn BAI3 conditional knockout (cKO) mice and infected them at days in vitro 4 (DIV4) with lentiviruses producing either active Cre recombinase or inactive mutant Cre (ΔCre, control). At DIV6, we additionally infected the cultures with lentiviruses expressing WT or mutant BAI3 for rescue experiments (fig. S1A). Last, at DIV9, we sparsely transfected the neurons with tdTomato and analyzed axonal growth, dendritic arborization, and soma size at DIV14. As described earlier (*20*), deletion of BAI3 in mixed neuron-glia cultures caused a massive increase in axon growth (a nearly threefold increase in axon length) and a less pronounced but notable increase in dendrite growth (~60% increase in dendrite length) (Fig. 2, A to F; and fig. S2, A to D). This marked phenotype in both axon and dendrite growth was fully rescued by WT BAI3, but not by any of the four mutant BAI3s (Fig. 2, A to F; and fig. S2, A to D). Compared with WT BAI3, most mutant constructs were expressed at similar levels in the mixed neuron-glia cultures, although the T4L mutant was expressed at slightly lower levels (fig. S1, B and C). None of the manipulations, the BAI3 deletions and various rescue conditions, had an effect on neuronal soma size (figs. S1D and S2E). In addition, deletion or overexpression of BAI3 had no effect on spine density and spine volume (fig. S3). These results suggest that the BAI3 GPCR activity is functionally important for regulating axonal and dendritic growth and that such regulation involves an ELMO-dependent mechanism. The fact that the two mutations designed to block ELMO binding had the same effect on dendritic and axonal growth (Fig. 2, A to F; and fig. S2, A to D) although they appeared to have somewhat different effects on BAI3’s GPCR activity (Fig. 1C) is most likely due to the noisy nature of GPCR activity measurements in transfected HEK293 cells.

**Fig. 3.**
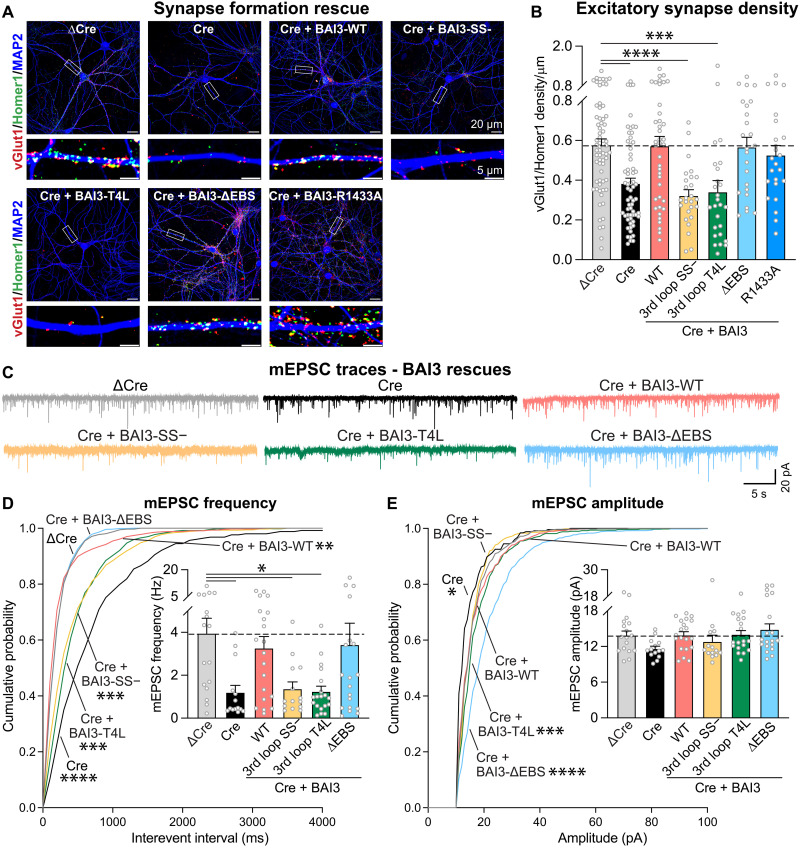
BAI3 GPCR signaling is essential for promoting synapse formation, whereas BAI3 ELMO binding is dispensable. (**A**) Representative overviews (top) and zoomed-in images of dendrites (bottom) of vGluT1- (red), Homer1- (green), and MAP2-stained neurons (blue). Control (ΔCre) and BAI3 KO neurons (Cre) were analyzed with or without lentiviral expression of the indicated BAI3 constructs. Experiments were performed in primary mixed neuron-glia cultures produced as described for Fig. 2A but subjected to immunocytochemistry instead of sparse transfections. (**B**) Summary graphs of the excitatory synapse density as a function of the expression of various BAI3 constructs. Double vGlurT1- and Homer1-positive puncta were quantified on primary dendrites (three dendrites per neuron). Note that the synapse density thus determined likely underestimates the synapse density because of its rigor, making it necessary to consider relative changes of test to controls as the key indicator of changes in synapse numbers. *N* (cells/experiments) = 66/8, 63/8, 40/5, 26/3, 26/3, 24/3, and 24/3 from left to right. (**C**) Representative miniature excitatory postsynaptic current (mEPSC) traces from hippocampal neurons in mixed neuron-glia cultures of BAI3 cKO mice obtained as described Fig. 2A but without sparse tdTomato transfections. (**D** and **E**) Cumulative distribution plots of the mEPSC interevent intervals (D) and amplitudes [(E); inset summary graphs of the mEPSC frequency (D) and amplitude (E)] recorded in hippocampal neurons as a function of WT and mutant BAI3 as described in (C). *N* (cells/experiments) = 17/4, 14/4, 20/4, 14/4, 19/4, and 19/4 from left to right. Data in (B), (D), and (E) are means ± SEM. Statistical significance was evaluated using one-way ANOVA with subsequent Dunnett’s multiple comparisons test for all bar graphs, or the Kolmogorov-Smirnov test for cumulative distributions in (D) and (E), comparing all conditions to the ΔCre controls (*P < 0.05, **P < 0.01, ***P < 0.001, and ****P < 0.0001).

In addition to restricting axonal and dendritic growth, BAI3 supports synapse formation (*20*, *22*, *24*, *33*–*35*). To test the effect of the mutations impairing GPCR activity or ELMO binding on synapse formation, we measured the synapse density in mixed neuron-glia cultures as a function of the expression of WT or mutant BAI3 (Fig. 3, A, and B, and fig. S2F). Notably, the GPCR activity mutations (T4L and SS−) abolished the ability of BAI3 to rescue the loss of synapses in BAI3-deficient cultures, whereas the two ELMO-binding mutations did not interfere with this ability (Fig. 3, A and B, and fig. S2F).

To further validate the conclusion that BAI3’s GPCR activity, but not its ELMO binding, is required for BAI3 function in synapse formation, we monitored spontaneous miniature excitatory postsynaptic currents (mEPSCs) using whole-cell patch-clamp recordings in neurons expressing WT or mutant BAI3 (Fig. 3C). mEPSCs are recordings of individual synaptic events, rendering them a proxy for both synapse numbers and synaptic function. The BAI3 deletion caused a large decrease (~75%) in the mean mEPSC frequency without affecting the mean mEPSC amplitude (Fig. 3, C to E), consistent with the decrease in synapse numbers (Fig. 3, A and B, and fig. S2F). The decrease in mEPSC frequency by the BAI3 deletion was fully rescued by WT BAI3 and by the ELMO-binding mutant of BAI3 (DEBS), but not by the two different GPCR activity-deficient mutants of BAI3 (Fig. 3, C and D). Cumulative probability plots of the interevent intervals confirmed the decrease in mEPSC frequency and the selective rescue of this phenotype by the ELMO-binding-deficient BAI3 mutant (Fig. 3D). These plots also uncovered a small change in mEPSC amplitude upon expression of GPCR-mutant BAI3 (Fig. 3, D and E). Most of these interventions had no effect on the intrinsic electrical properties of neurons, except that the T4L mutant rescue condition exhibited a slight decrease in membrane capacitance (fig. S4, A1 and A2).

**Fig. 4.**
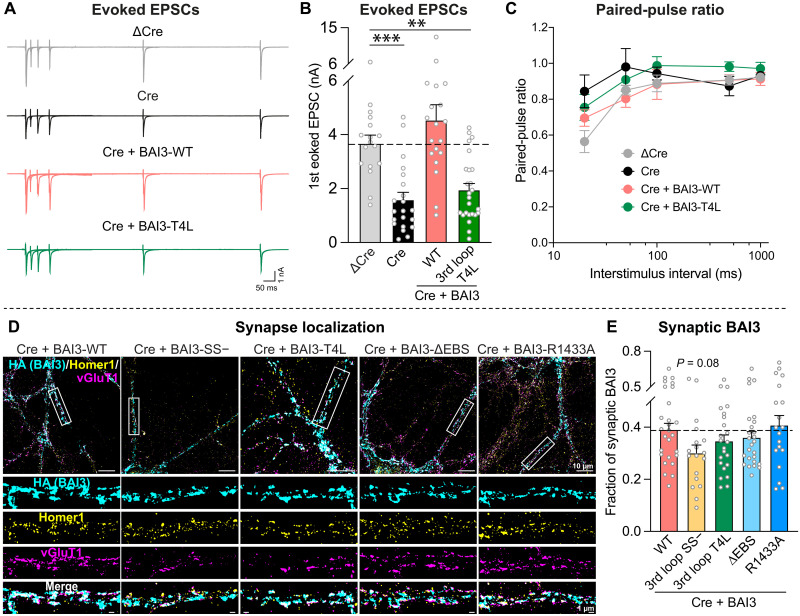
BAI3 GPCR signaling is essential for promoting synaptic transmission, whereas BAI3 ELMO binding is dispensable. (**A**) Representative traces of evoked EPSCs monitored in hippocampal neurons as a function of the expression of WT or mutant BAI3 as described in Fig. 3A, without immunocytochemistry. EPSCs were evoked by closely spaced pairs of action potentials elicited by extracellular stimulation with a concentric electrode. (**B**) Summary graph of the amplitude of the first evoked EPSC during paired-pulse stimulation for the experiments shown in (A). *N* (cells/experiments) = 17/5, 21/5, 19/5, and 25/5 from left to right. (**C**) Summary plot of the paired-pulse ratios of evoked EPSC amplitudes as a function of the interstimulus interval for the experiments depicted in (A). (**D**) STED super-resolution microscopy images illustrating the localization of HA-tagged WT or mutant BAI3 (cyan) relative to that of synapses identified by staining for vGlut1 (magenta) and Homer1 (yellow). Neurons were obtained as described for Fig. 3A but stained and imaged by STED microscopy to determine whether mutant BAI3 proteins are localized to synapses. (**E**) Summary graph of the fraction of exogenously expressed HA-tagged BAI3 proteins that are colocalized with both vGlut1 and Homer1. *N* (cells/experiments) = 27/3, 18/3, 23/3, 26/3, and 19/3 from left to right. Data in (B), (C), and (E) are means ± SEM. Statistical significance was evaluated using one-way ANOVA with subsequent Dunnett’s multiple comparisons test for all bar graphs or two-way ANOVA for summary plot in (C), comparing all conditions to the WT or ΔCre controls (**P < 0.01 and ***P < 0.001).

The fact that the mEPSC phenotype is larger than the synapse density phenotype (Fig. 3, A to E, and fig. S2F) could be explained by at least two hypotheses. First, it is possible that the mEPSC measurements are more accurate and that the synapse density measurements underestimate the phenotype, which is plausible given the rigorous approach we used to measure synapse densities that only includes “puncta,” which are positive for both a pre- and a postsynaptic marker. Second, it is conceivable that the BAI3 deletion affects not only synapse numbers but also synapse function, which again is plausible given that, at least, for teneurin-3/4 deletions, both synapse numbers and synapse functions were impaired (*44*). To differentiate between these two possibilities, we performed for selected constructs evoked EPSC recordings using a paired-pulse stimulation paradigm that assesses the presynaptic release probability (Fig. 4, A to C). We measured both the amplitude of the first EPSC (Fig. 4B) and the ratio of the second to the first EPSC amplitudes in dependence of the interstimulus interval (Fig. 4C). As expected, the first EPSC amplitude, a measure of synaptic connectivity as the sum of synapse numbers and synaptic function, was suppressed by the BAI3 deletion, rescued with WT BAI3, but not rescued with T4L-mutant BAI3 that exhibits an impaired GPCR activity (Fig. 4B). However, none of the BAI3 manipulations significantly altered the paired-pulse ratio, although there was a tendency toward an increase in this ratio upon deletion of BAI3, suggestive of a small decrease in release probability (Fig. 4C). Moreover, none of these interventions had any effect on the intrinsic electrical properties of neurons (fig. S4, B1 and B2). Together, these results suggest that the BAI3 deletion produces primarily a decrease in synapse numbers, which can be rescued by BAI3 proteins with a mutated ELMO binding site, but not by BAI3 proteins with impaired GPCR activity.

It is possible that the GPCR and ELMO-binding mutations alter the localization of exogenously expressed BAI3. To address this possibility, we performed stimulated emission depletion (STED) super-resolution microscopy (Fig. 4, D and E). We found that the exogenous BAI3 largely localized to synapses in a manner that was unaffected by the various BAI3 mutations (Fig. 4E). Thus, it seems unlikely that the effect of the GPCR-activity or ELMO-binding mutations on synapse numbers or lack thereof was mediated by a change in the BAI3 localization induced by these mutations.

### The BAI3 tethered agonist is neither sufficient nor required for BAI3 functions

**Fig. 5.**
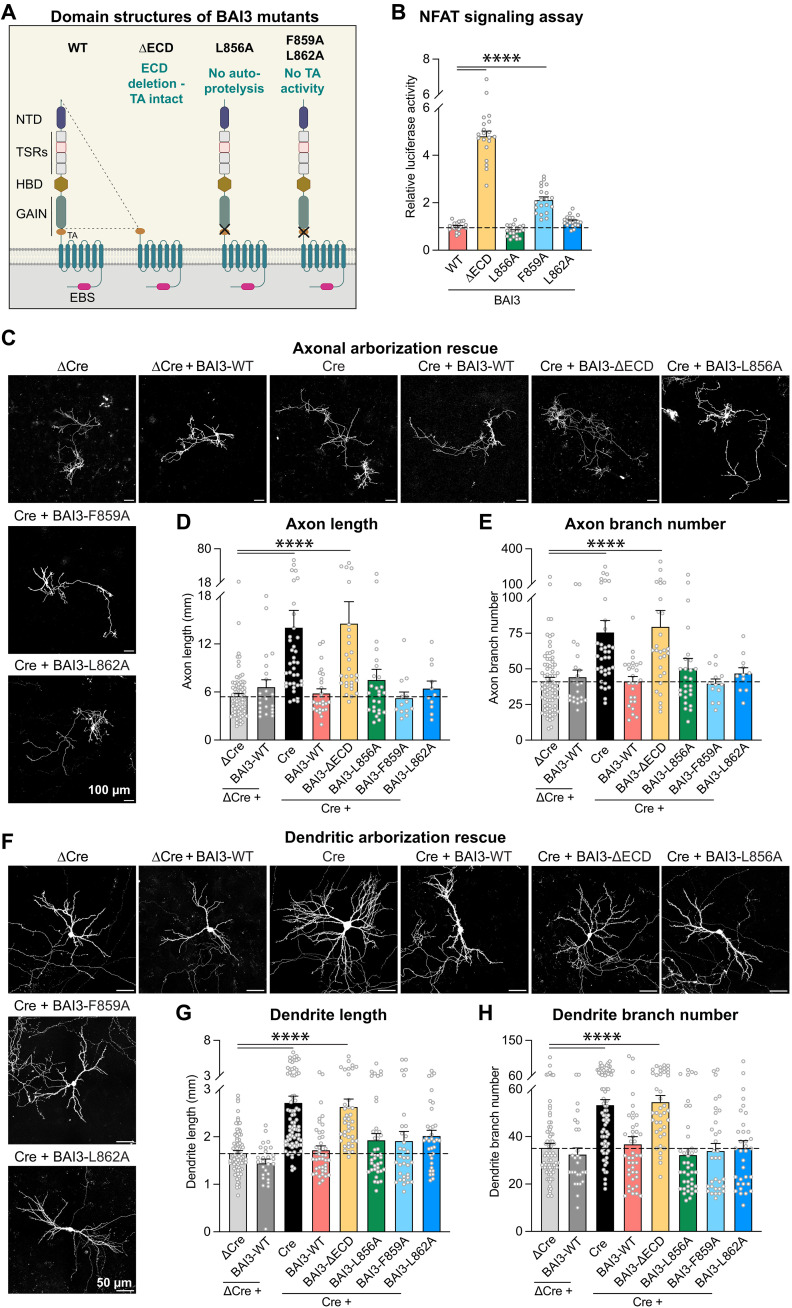
Exposure of the tethered agonist (“Stachel”) of BAI3 by deletion of the BAI3 ECDs activates BAI3’s GPCR activity but abolishes BAI3’s function in restricting axonal and dendritic arborizations, whereas mutating the tethered agonist of BAI3 has no effect. (**A**) Domain structures of WT BAI3 and of BAI3 with mutations deleting BAI3’s ECDs [ΔECD; exposes BAI3’s tethered agonist (TA)], blocking autoproteolysis (L856A), or impairing tethered agonist binding (F859A or L862A). Created in BioRender. Y. Miao (2026), https://BioRender.com/f6kp3kp. (**B**) The BAI3 ECD deletion (ΔECD) activates BAI3’s GPCR activity monitored using NFAT transcriptional reporter assays, whereas blocking BAI3’s GAIN-domain autoproteolysis or mutating its tethered agonist has no effect on BAI3’s GPCR activity. *N* (samples/experiments) = 20/4. (**C** to **H**) Representative images of cultured hippocampal neurons sparsely transfected for quantifications of the effect of BAI3 mutations on the length and branching of axons (C) or dendrites (F) as described for Fig. 2 (A and D), respectively, and summary graphs of length [(D) and (G)] and branch numbers [(E) and (H)] of axons [(D) and (E)] and dendrites [(G) and (H)] as a function of the expression of WT or mutant BAI3 [(D) and (E): *N* (cells/experiments) = 79/20, 22/10, 37/14, 26/8, 28/9, 26/8, 13/6, and 11/7 from left to right; (G) and (H): *N* (cells/experiments) = 82/12, 27/6, 79/12, 42/7, 40/4, 41/9, 32/7, and 34/6 from left to right]. Data in (B), (D), (E), (G), and (H) are means ± SEM. Statistical significance was determined using one-way ANOVA followed by Dunnett’s multiple comparisons test comparing all conditions to the WT or ΔCre controls (****P < 0.0001). Please note that WT group samples in (B) are the same as those in Fig. 1C. ΔCre, Cre, and Cre + BAI3-WT groups in (D), (E), (G), and (H) have overlapping or same samples as those in Fig. 2 (B, C, E, and F) (see Materials and Methods for details).

To investigate whether BAI3’s tethered agonist (that is released by cleavage when the GAIN domain undergoes autoproteolysis) regulates BAI3 functions, we generated a deletion mutant that lacks the ECDs of BAI3 up to the tethered agonist (ΔECD). In the ΔECD mutant of BAI3, the tethered agonist is constitutively exposed, as would occur if the ECDs were mechanically dissociated from the GPCR moiety (Fig. 5A). In addition, we constructed point mutations that disrupt BAI3’s autoproteolysis (L856A) or interfere with the binding of the tethered agonist into the GPCR binding pocket (F859A or F862A) (Fig. 5A). Analysis of the GPCR activity of these mutants, using the NFAT reporter assay in transfected HEK293T cells, demonstrated that the ECD deletion exposing the tethered agonist massively (~5-fold) activated the BAI3 GPCR activity (Fig. 5B). Two of the three tethered agonist–related point mutations (L856A and L862A) had no effect on GPCR activity, whereas the third tethered agonist–related point mutation (F859A) modestly activated the GPCR activity (Fig. 5B). Our results are consistent with previous studies suggesting that ECD deletions in adhesion GPCRs greatly enhance their constitutive activity (*11*, *12*), but differ from a recent systematic study which failed to demonstrate this for BAI3 (*43*) for reasons that we now do not understand.

Does constitutive elevation of the BAI3 GPCR activity by the ECD deletion or, conversely, disruption of the BAI3 tethered agonist influence its physiological role in controlling axonal and dendritic arborizations? Rescue experiments revealed that the ECD deletion completely abolished the ability of BAI3 to reverse the exuberant axonal and dendritic arborization induced by the BAI3 deletion, demonstrating that simply stimulating BAI3’s constitutive GPCR activity is functionally insufficient for controlling axonal and dendritic growth (Fig. 5, C to H; and fig. S6, A to D). Although one might have expected that the ΔECD deletion mutant with its massive intrinsic GPCR activity acts as a dominant positive, i.e., suppresses axonal and dendritic growth below WT levels, the BAI3 mutant lacking ECDs simply had no effect (Fig. 5, C to H; and fig. S6, A to D). Conversely, the tethered agonist point mutations that are designed to block the GAIN-domain autoproteolysis or the tethered agonist insertion into the GPCR ligand-binding pocket (L856A, F859A, and L862A) were fully able to rescue the BAI3 deletion phenotype (Fig. 5, C to H; and fig. S6, A to D). Compared to WT BAI3, the F859A and L862A mutants were expressed at similar levels, whereas L856A was expressed at a slightly higher levels and the ΔECD deletion mutant was expressed at modestly lower levels (fig. S5, A and B). None of these mutations affected neuronal soma size (figs. S5C and S6E). Thus, the tethered agonist likely does not control BAI3 function in regulating axonal and dendritic arborizations, and simply activating BAI3 GPCR signaling does not regulate axonal or dendritic arbors.

**Fig. 6.**
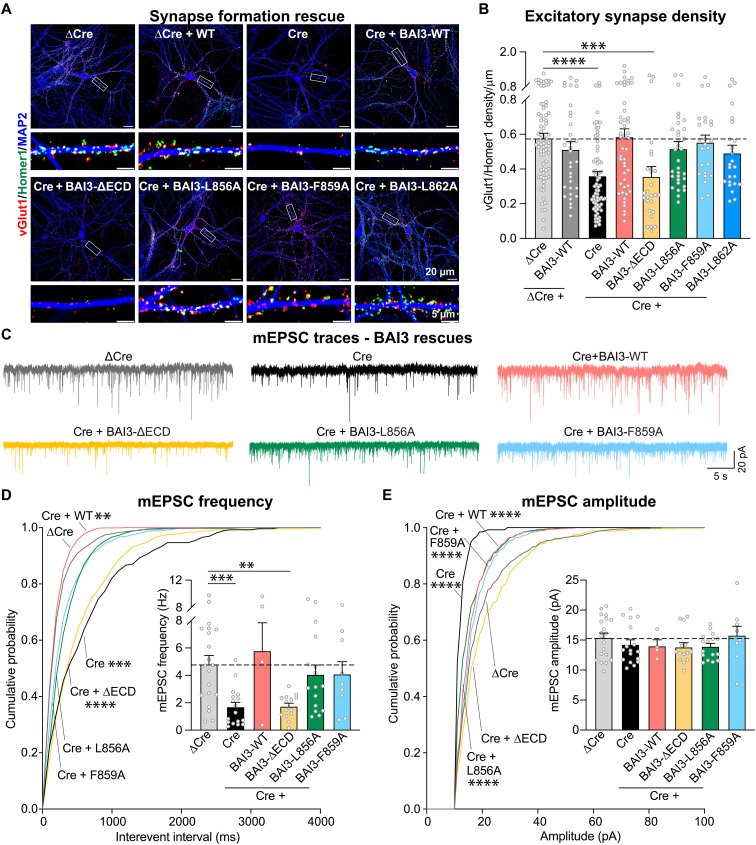
Activation of GPCR signaling by BAI3 via its tethered agonist is neither sufficient nor required for synapse formation. (**A**) Representative overviews (top) and zoomed-in dendrite images (bottom) of neurons stained for the pre- and postsynaptic markers vGluT1 (red) and Homer1 (green), respectively, and for the dendritic marker MAP2 (blue). Control (ΔCre) or BAI3 KO neurons (Cre) were analyzed with or without lentiviral expression of the indicated BAI3 constructs. Experiments were performed in primary mixed neuron-glia cultures produced as described for Fig. 2A except that the cultures were subjected to immunocytochemistry instead of sparse transfections. (**B**) Summary graphs of excitatory synapse density as a function of the expression of various BAI3 constructs. Synapse densities were quantified as the density of double-positive vGlut1 and Homer1 puncta on primary dendrites as described for Fig. 3B. *N* (cells/experiments) = 74/9, 32/4, 71/9, 48/6, 27/3, 32/4, 24/3, and 24/3 from left to right. (**C**) Representative mEPSC traces from hippocampal neurons in mixed neuron-glia cultures of BAI3 cKO mice obtained as described in Fig. 2A but without sparse tdTomato transfections. (**D**) Cumulative distribution plot of the mEPSC interevent intervals and summary graph of the mEPSC frequency (inset) recorded in hippocampal neurons as a function of the expression of WT and mutant BAI3 for experiments in (C). *N* (cells/experiments) = 20/6, 17/6, 4/2, 14/4, 16/6, and 9/3 from left to right. (**E**) Cumulative distribution plot and summary graph (inset) of the mEPSC amplitude for the experiments shown in (C). Data in (B), (D), and (E) are means ± SEM. Statistical significance was evaluated using one-way ANOVA with subsequent Dunnett’s multiple comparisons test for all bar graphs or the Kolmogorov-Smirnov test for cumulative distributions in (D) and (E), comparing all conditions to the ΔCre controls (**P < 0.01; ***P < 0.001; ****P < 0.0001).

**Fig. 7.**
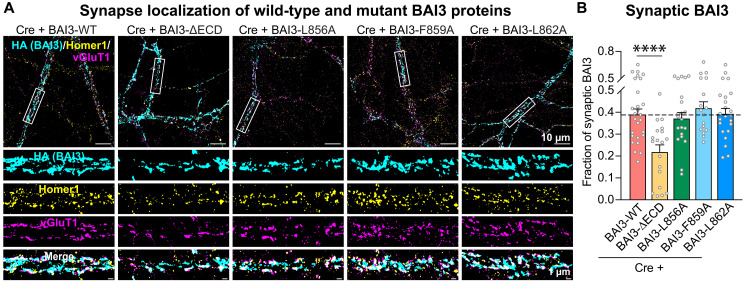
Localization of WT and mutant BAI3 proteins at synapses. (**A**) STED super-resolution microscopy images illustrating the localization of HA-tagged WT or mutant BAI3 (cyan) relative to that of synapses identified by staining for vGlut1 (magenta) and Homer1 (yellow). Neurons were obtained as described for Fig. 6A but were stained and imaged by STED microscopy to determine whether mutant BAI3 proteins are localized to synapses. (**B**) Summary graph of the fraction of exogenously expressed WT and mutant HA-tagged BAI3 proteins that are colocalized with both vGlut1 and Homer1. *N* (cells/experiments) = 27/3, 19/3, 21/3, 17/3, and 23/3 from left to right. Data are means ± SEM. Statistical significance was evaluated using one-way ANOVA with subsequent Dunnett’s multiple comparisons test comparing all groups to the WT controls (****P < 0.0001).

We next analyzed the role of the tethered agonist in BAI3’s function of promoting synapse formation and synaptic transmission (Fig. 6, A to E, and fig. S6F). Again, the ECD deletion abolished the ability of WT BAI3 to rescue the large decrease in synapse density and spontaneous synaptic transmission events (mEPSCs) induced by the BAI3 deletion (Fig. 6, A to E, and fig. S6F). The point mutations blocking GAIN-domain autoproteolysis or altering the BAI3 tethered agonist sequence, however, had no effect on the rescue (Fig. 6, A to E, and fig. S6F). None of these interventions altered the intrinsic electrical properties of neurons (fig. S7, A and B). Moreover, the tethered agonist mutation F859A did not alter the ability of BAI3 to fully rescue the deficit in evoked EPSC amplitude produced by the BAI3 deletion (fig. S7, C and D). No significant changes in the paired-pulse ratio or passive electrical properties were observed upon all manipulations (fig. S7, E to G). Thus, the tethered agonist of BAI3 is dispensable for synapse formation and for synaptic function. Super-resolution STED microscopy revealed that BAI3 mutants L856A, F859A, and L862A are localized to synapses similarly to WT BAI3, whereas the ΔECD BAI3 mutant showed a reduction in synaptic localization (Fig. 7, A and B). These results suggest that BAI3 facilitates synaptogenesis and enhances excitatory synaptic transmission via its ECDs’ activities that are critical for synaptic localization, but these processes do not depend on autoproteolysis or tethered agonist activity. Together, our experiments, therefore, indicate that activating BAI3 GPCR activity by itself does not activate its physiological functions without the BAI3 ligand-binding domains, whereas impairing tethered agonist–mediated signaling by BAI3 has no effect on its physiological functions.

### The HBD and GAIN domains of BAI3 are required for NFAT signaling, but not for axon and dendritic arborizations or synaptic transmission

**Fig. 8.**
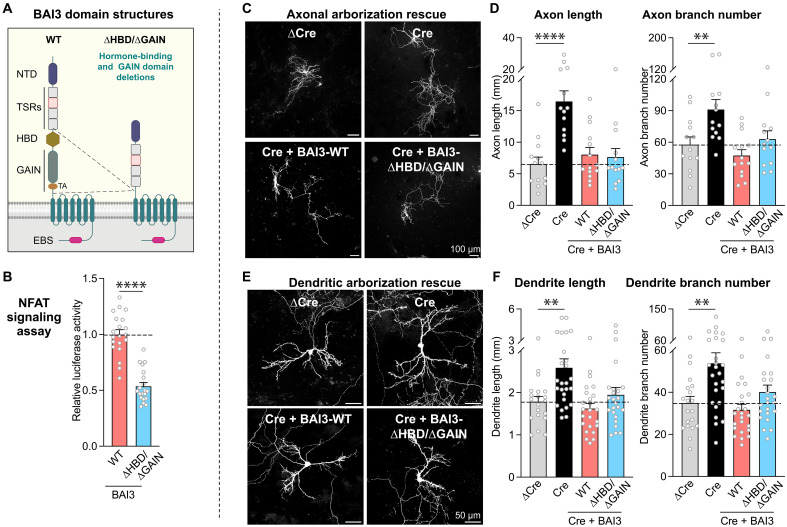
The HBD and GAIN domain of BAI3 are dispensable for BAI3-dependent regulation of axonal and dendritic growth. (**A**) Domain structures of WT BAI3 and of BAI3 with a deletion of the HBD and GAIN domain (ΔHBD/ΔGAIN). Created in BioRender. Y. Miao (2026), https://BioRender.com/f6kp3kp. (**B**) BAI3 with a deletion of the hormone binding and GAIN domains (ΔHBD/ΔGAIN) exhibits reduced GPCR-mediated NFAT signaling activity as assayed using a transcriptional reporter in transfected HEK293T cells. *N* (samples/experiments) = 20/4. (**C**) Representative images of cultured hippocampal neurons used for quantifications of the effect of BAI3 mutation on axon length and axon branching. Experiments were carried out as described for Fig. 2A. (**D**) Summary graphs of the length (left) and branch numbers of axons (right) as a function of the expression of WT or mutant BAI3 for experiments in (C). *N* (cells/experiments) = 13/4, 13/4, 14/3, and 13/4 from left to right. (**E**) Representative images illustrating dendrite length and branching in hippocampal neurons from mixed neuron-glia cultures of BAI3 cKO mice. Experiments were carried out as described for Fig. 2D. (**F**) Summary graphs of the length (left) and branch numbers (right) of dendrites as a function of the expression of WT or mutant BAI3 for experiments in (E). *N* (cells/experiments) = 20/5, 26/5, 24/5, and 24/5 from left to right. All numerical data are means ± SEM. Statistical significance was determined using unpaired two-tailed Student’s *t* tests in (B) (****P < 0.0001) and one-way ANOVA followed by Dunnett’s multiple comparisons test in (D) and (F), comparing all groups to the ΔCre controls (**P < 0.01 and ****P < 0.0001).

The expendability of BAI3’s tethered agonist for BAI3’s functions is unexpected given the conserved presence of a tethered agonist in the canonical GAIN domains of all adhesion GPCRs. Our results prompt the question whether the conserved GAIN domain of BAI3 has any role in its physiological functions. To address this question, we analyzed the effect of a BAI3 deletion that removes both its GAIN domain and its HBD (ΔHBD/ΔGAIN) (Fig. 8A). This double deletion was examined instead of a deletion of only the GAIN domain because BAI3 mutants lacking only the GAIN domain appeared to exhibit poor surface expression, whereas the double HBD/GAIN-domain deletion did not interfere with the surface transport of BAI3; its expression was slightly higher than that of WT BAI3 (fig. S8, A and B). Signal transduction assays in transfected HEK293T cells showed that the removal of the HBD and GAIN domain modestly decreased NFAT signaling (Fig. 8B), suggesting that the GAIN domain contributes to the full GPCR activity of BAI3.

We then performed rescue experiments similar to those described above. Unexpectedly, the HBD/GAIN-domain deletion did not have any negative effect on the ability of BAI3 to rescue the exuberant axonal and dendritic arborizations caused by the BAI3 deletion (Fig. 8, C to F). Thus, the BAI3 HBD and GAIN domains are involved in BAI3-mediated GPCR signaling as analyzed using NFAT-mediated transcriptional activation assay (Fig. 8B) but are functionally dispensable for BAI3’s function in controlling axonal and dendritic growth (Fig. 8, C to F). In addition, none of these manipulations had any effect on soma area size (fig. S8C).

**Fig. 9.**
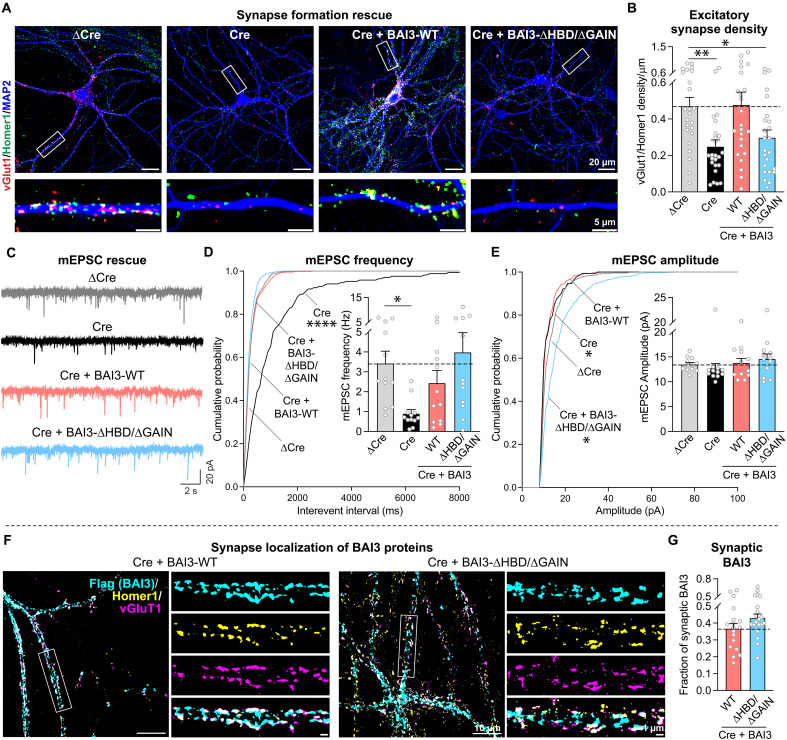
The HBD and GAIN domain of BAI3 contribute to synapse formation but are dispensable for synapse function. (**A**) Representative overviews (top) and zoomed-in dendritic images (bottom) of vGluT1- (red), Homer1- (green), and MAP2-stained neurons (blue). Control (ΔCre) or BAI3 KO neurons (Cre) were analyzed with or without lentiviral expression of the indicated BAI3 constructs. Experiments were performed as in Fig. 2A but used immunocytochemistry instead of sparse transfections. (**B**) Summary graphs of excitatory synapse density as a function of various BAI3 constructs. Synapses were quantified as the density of double-positive vGlut1 and Homer1 puncta on primary dendrites (see Fig. 3B. *N* (cells/experiments) = 24/3, 24/3, 24/3, and 24/3 from left to right. (**C**) Representative mEPSC traces from hippocampal neurons. Mixed neuron-glia cultures from BAI3 cKO mice were infected with ΔCre or Cre lentiviruses at DIV4 and BAI3 constructs lentiviruses at DIV6 and analyzed at DIV14. (**D** and **E**) Cumulative distribution plots of mEPSC interevent intervals (D, left) or amplitudes (E, left) and summary graphs of the mEPSC frequency [(D), right] and amplitudes [(E), right] for the experiments shown in (C). *N* (cells/experiments) = 12/3, 12/3, 12/3, and 12/3 from left to right. (**F**) STED images of neurons in BAI3-deficient hippocampal neuron-glia cultures expressing the indicated BAI3 constructs and stained by immunocytochemistry [Flag-tagged BAI3 (cyan), Homer1 (yellow), and vGlut1 (magenta)]. (**G**) Quantification of the colocalization of Flag-tagged BAI3 proteins with synaptic markers vGlut1 and Homer1 for the experiments in (F). *N* (cells/experiments) = 18/3 and 20/3 from left to right. Data are means ± SEM. Statistical significance was determined using unpaired two-tailed Student’s *t* tests (G), one-way ANOVA followed by Dunnett’s multiple comparisons test [(B), (D), and (E)], or Kolmogorov-Smirnov tests for cumulative distributions [(D) and (E)], comparing all conditions to ΔCre controls (*P < 0.05, **P < 0.01, and ****P < 0.0001).

Do the HBD and GAIN domains of BAI3 contribute to synapse formation, although they are not required for BAI3’s function in regulating axonal and dendritic growth? Rescue experiments measuring the synapse density in BAI3-deficient mixed neuron-glia cultures as a function of the expression of WT or HBD/GAIN-domain deleted BAI3 suggested that the HBD/GAIN-domain deletion partly impaired the ability of BAI3 to promote synapse formation (Fig. 9, A and B). Measurements of mEPSCs using whole-cell patch-clamp recordings, however, showed that BAI3 lacking the HBD and GAIN domains completely rescued the large decrease in mEPSC frequency of BAI3-deficient neurons (Fig. 9, C to E). None of these manipulations had any effect on the intrinsic electrical properties of neurons (fig. S8, D and E). These results suggest that the BAI3 HBD and GAIN domains are not essential for the promotion of synaptic function by BAI3 but may contribute to synapse formation. The differences in the results produced by the two assays for synapses used here, measurements of synapse density using immunocytochemistry and measurements of synaptic connectivity using mEPSC recordings, is not due to a change in the synaptic localization of BAI3 by the HBD/GAIN-domain deletion because STED super-resolution microscopy showed that WT and HBD/GAIN domain–deficient BAI3 equally localized to synapses (Fig. 9, F and G).

## DISCUSSION

**Fig. 10.**
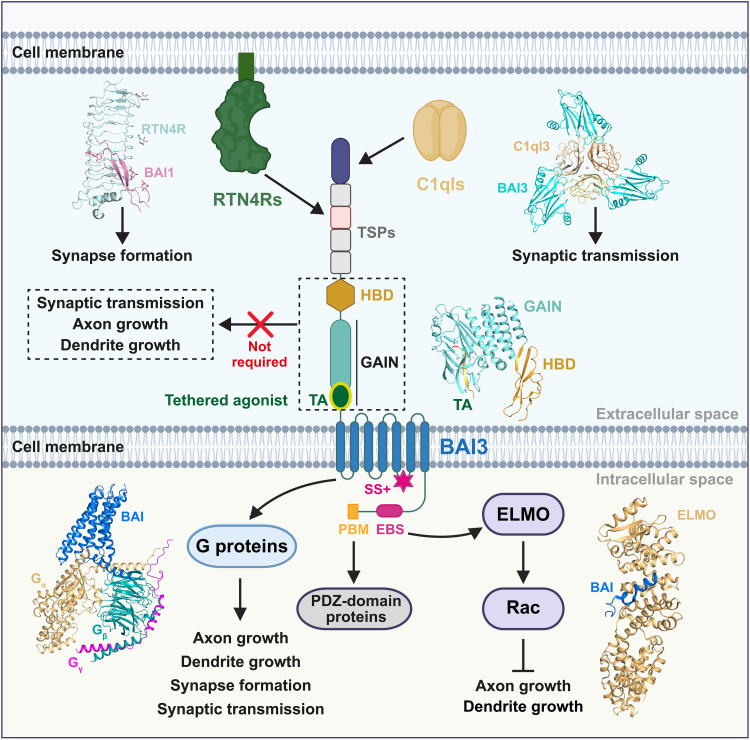
Schematic of the interactions and functions mediated by BAI3 that acts as an adhesion GPCR to control the development of axonal and dendritic arbors and the formation of synapses independent of its tethered agonist. BAI3 is a constitutively active adhesion GPCR that interacts extracellularly with at least two ligands implicated in circuit development: RTN4Rs and C1qls (*21*, *25*). Intracellularly, BAI3 recruits G protein complexes and probably arrestins and additionally binds to ELMO proteins that function as Rac–guanosine triphosphatase–activating proteins and to PDZ-domain proteins such as PSD95 that form postsynaptic specializations (*28*, *30*). Via these interactions, BAI3 performs at least three mechanistically distinct functions, restricting axon growth, restricting dendrite growth, and promoting synapse formation by acting as a GPCR. Now dominant models propose that adhesion GPCRs are activated via binding of a “tethered agonist,” but our results reveal that none of the three identified functions of BAI3 is dependent on its tethered agonist and that coupling to ELMO signaling is only required for regulating axon and dendrite growth but not for synapse formation. Thus, BAI3 is involved in multiple aspects of neuronal development through distinct molecular mechanisms. The structures in this figure were generated from Alpha Fold (BAI3–G protein) (*53*) or Protein Data Bank IDs: 6IDX (ELMO2-BAI) (*29*), 7R86 (RTN4R-BAI1) (*25*), 8VWY (C1ql3-BAI3) (*23*), and 4DLO (BAI3 GAIN domain) (*4*). PBM denotes the PDZ-binding motif. Created in BioRender. Y. Miao (2026), https://BioRender.com/2ei9ono.

Adhesion GPCRs are thought to be activated via their tethered agonist that is embedded in their canonical GAIN domain. The tethered agonist is exposed when the ECDs of adhesion GPCRs are mechanically dissociated from their C-terminal GPCR moiety (*5*, *7*–*9*, *45*) but may also be freed by partial unfolding of the GAIN domain without being totally dissociated from it, for example, if GAIN-domain cleavage does not occur as hypothesized for BAI3 (*16*). We here tested the tethered agonist hypothesis on the example of BAI3, a brain-specific adhesion GPCR (*17*). Our previous results showed that, consistent with earlier studies (*22*, *28*, *33*, *34*), BAI3 performs three independent functions when analyzed in mixed neuron-glia cultures, namely, the restriction of axon growth and of dendritic arborizations and the promotion of synapse formation (*20*). Based on these results, we examined the mechanism of action of BAI3 as an adhesion GPCR, asking specifically whether (i) BAI3 acts as a GPCR, as an adhesion molecule, or both; (ii) BAI3’s tethered agonist stimulates its GPCR activity; (iii) the tethered agonist–mediated stimulation of BAI3 constitutes the mechanism of action of BAI3 in the three functions we examined; (iv) the canonical GAIN domain that harbors the tethered agonist performs additional essential functions independent of the tethered agonist; and (v) coupling of BAI3 to ELMO-mediated Rac-GEF activity is essential for various BAI3 functions. Based on the evidence discussed below, our results indicate that the BAI3 GAIN domain, in general, and the BAI3 tethered agonist in particular are not required for BAI3’s functions in controlling axon and dendrite growth and in promoting synaptic transmission, whereas the BAI3 GPCR activity is absolutely essential for these BAI3 functions, and that ELMO-mediated Rac signaling is likely involved in the BAI3-mediated regulation of axonal and dendritic arborizations but is dispensable for synapse formation (Fig. 10).

The evidence for these conclusions is as follows. First, impairing the GPCR activity of BAI3 by two different mutations, the T4L insertion into the third cytoplasmic loop or the deletion of a short sequence from the third cytoplasmic loop that mimics a naturally occurring splice variant, abolished the ability of WT BAI3 to rescue the BAI3-deletion phenotypes in axonal and dendritic arborization and synapse formation (Figs. 1 to 4). Second, mutating the EBS of the BAI3 cytoplasmic tail blocked the ability of BAI3 to reverse the exuberant axonal and dendritic arborization present in BAI3-deficient cultures but had no effect on synapse formation (Figs. 1 to 4). Third, deleting the ECDs of BAI3 with full retention of the tethered agonist strongly activated its GPCR activity but abolished BAI3’s functions in dendritic and axonal growth and in synapse formation (Figs. 5 to 7). This mutant did not produce a dominant effect but was still at least partly localized to synapses. Thus, expressing a constitutively activated BAI3 GPCR on its own has no major functional effect in cultured neurons. Fourth, mutating the tethered agonist of BAI3 did not impair the ability of BAI3 to rescue either the axonal or dendritic arborization phenotypes or the defect in synapse formation observed in BAI3-deficient cultures (Figs. 5 to 7). Last, even the deletion of the entire HBD and GAIN domains did not alter the ability of BAI3 to control axonal and dendritic growth in neurons, although it did have a partial effect on synapse formation (Figs. 8 and 9).

It is unclear whether BAI3’s GAIN domain undergoes autoproteolysis because, in transfected HEK293 cells and as a recombinant protein, the BAI3 GAIN domain lacks autoproteolysis, although BAI3 autoproteolysis may occur in brain (*4*, *16*). To account for the possibility that BAI3’s tethered agonist activates BAI3 function by a mechanism not requiring autoproteolysis of the GAIN domain (for example, by a looping out of the relevant peptide sequence or by partial unfolding of the GAIN domain), we analyzed not only a BAI3 mutant that blocks autoproteolysis (L856A) but also two BAI3 point mutations that change the highly conserved sequence of the tethered agonist in a manner expected to abolish tethered agonist–mediated GPCR activation (F859A and L862A) (Figs. 5 to 7). Moreover, we analyzed a BAI3 mutant in which the entire GAIN domain, including the tethered agonist and the HBD, was deleted, which excludes all possibilities of a tethered agonist action (Fig. 8 and 9). All of these mutations failed to block the functions of BAI3 in controlling axonal and dendritic growth and in promoting synapse formation, excluding an essential role for the BAI3 tethered agonist in these functions of BAI3.

As is often observed with unexpected findings, our results raise further questions. First, what is the mechanism of action of BAI3 as a GPCR? Its signaling pathways, the specific G proteins and effector mechanisms used, are likely highly organized spatially, but how does this work? The synaptic concentration of BAI3 (Figs. 4, D and E; 7, A and B; and 9, F and G) suggests a localized signal transduction mechanism similar to that described for latrophilins (*46*, *47*), but the molecular details remain unknown.

Second, what is the role of the ELMO-dependent Rac signaling pathway in axonal and dendritic arborizations, and why are both the BAI3 GPCR activity and BAI3 ELMO binding required for regulating axonal and dendritic arborizations? In BAI1, ELMO binding does not appear to be essential for dendritic growth control (*37*), whereas, in our experiments, using two different mutations ELMO binding was clearly required for controlling both axonal and dendritic growth. It is tempting to speculate that BAI3 restricts axon and dendrite growth via activation of Rho/Rac–guanosine triphosphatases that is mediated by ELMO, but further experiments are needed to map out the molecular pathways involved.

Third, how is ligand binding to BAI3 (especially RTN4R binding) connected to its GPCR activity? We previously showed that RTN4R binding to BAI3 is essential for all of its functions, but it is unclear whether such binding acts to localize BAI3 to specific sites, particularly synapses, or whether it regulates the activity of BAI3’s GPCR moiety and possibly Rho/Rac signaling.

Fourth, what is the relation of BAI3 to other synaptic adhesion GPCRs, i.e., to other BAI isoforms and to latrophilins? BAI1 and BAI3 deletions have the same robust phenotypes, indicating that they are not functionally redundant (*20*). Previous studies on BAI1 revealed similar loss-of-function phenotypes as we characterized for BAI3 and suggested that ELMO binding by BAI1 is not required for promoting synapse formation (*38*) or regulating dendritic growth (*37*). Latrophilin deletions only affect synapse numbers, but not dendritic and axonal arborizations, indicating a different mechanism of action (*46*, *47*). The fact that the loss of synapses after latrophilin deletions does not automatically lead to a change in axonal and dendritic arborizations also demonstrates that these phenotypes of the BAI3 deletion are independent of each other. The distinct roles of the different adhesion GPCRs are truly intriguing and arguably merit further study.

Last, what is the function of BAI3 in the developing versus the adult brain and in neurons versus glia? The present study purposely did not differentiate between neuronal and glial BAI3 functions, but previous studies suggested that the axonal arborization is regulated by glial BAIs whereas the dendritic arborization is controlled by neuronal BAIs (*25*). Given that synapses are being continuously restructured throughout life and that BAI3 is highly expressed in mature brain, it seems likely that the regulation of axonal and dendritic arborizations described here is operating throughout life, but as described above, the mechanisms involved remain uncertain.

In addition to raising these questions, our experiments involve inherent limitations. A major limitation of our study is that it examines the structure-function relations of BAI3 only in cultured neurons, not in vivo, thus using cultured neurons as a reduced system for analyses of neuronal development and synapse formation as such. In the three-dimensional context of the brain, BAI3’s functions may be more complex and multifaceted. Moreover, we studied only neurons derived from one heterogeneous brain region, the hippocampus, and assessed only three parameters, axonal and dendritic arborizations, and synaptic connectivity, because we previously found that these were altered by the deletion of BAI3 (*20*). In addition, we supplemented the examination of the three main parameters with analyses of soma size and passive electrical properties. Thus, we cannot exclude the possibility that BAI3 may have other functions that are mediated by other mechanisms and/or may apply to specific neurons and synapses in the brain in vivo. Synapses in the brain are diverse and likely dependent on distinct sets of synaptic adhesion molecules with different roles. It is therefore possible that the precise complement of synaptic adhesion molecules in different types of synapses may differentially affect the structure-function relations of BAI3 in these synapses in vivo and that, in some in vivo contexts, the GAIN domain of BAI3 may be functionally essential or its GPCR activity may be dispensable. This possibility can only be tested by investigating a range of synapses in vivo using structure-function studies, an endeavor that will require extensive future studies that are beyond the scope of the current project. Our experiments were specifically designed to define the signaling mechanisms of BAI3 in developing cultured neuron and functional synapses formed by these neurons as a reduced system, and our various conclusions may thus not always apply to an in vivo context. Last, we only tested a limited number of BAI3 mutations and protein interactions; for example, BAI3 may interact with additional cytoplasmic proteins as described for BAI1 (*48*–*50*) that are likely functionally important. Again, this possibility clearly merits further investigation in future studies.

In summary, we demonstrate that in neuron-glia cultures, BAI3 regulates the growth of axons and dendrites and the formation of synapses in cultured neurons by a mechanism that requires its GPCR activity but is independent of its GAIN domain and tethered agonist. Although we also document that the tethered agonist of BAI3 strongly activates BAI3’s GPCR activity, our results show that, at least for this particular adhesion GPCR and for the particular functions examined here, the tethered agonist is not an essential regulator of its biological functions, whereas BAI3’s GPCR activity is absolutely required. Last, we report a mechanistic difference between the BAI3 functions in regulating axon and dendrite growth versus promoting synapse formation in that the former, but not the latter, requires BAI3’s EBS in its cytoplasmic region.

## MATERIALS AND METHODS

### BAI3 cKO mice

As detailed in our previous study (*25*), BAI3 cKO mice were provided by M. Yuzaki from Keio University (*22*) and were crossed with CD1 IGS (International Genetic Standardization) WT mice (Charles River Laboratories, 022) to enhance breeding efficiency and create a hybrid genetic background. The primers used for genotyping BAI3 cKO mice are provided below:

1) BAI3 set 1 forward: TCT CAG TCT TCA TGG AGT GG

2) BAI3 set 1 reverse: TCT GTG GTG CAA GGA ACC AT

3) BAI3 set 2 forward: AAAGCAAGGAGGCGTATCCAG

4) BAI3 set 2 reverse: GGCAGGATGTGCTAAGCAAATG

Quantitative reverse transcription polymerase chain reaction (PCR) validation of the BAI3 cKO was performed in the previously published paper (*20*) as the experiments in both manuscripts were conducted concurrently. All animal procedures received approval from the Stanford Institutional Animal Care and Use Committee and the Administrative Panel on Laboratory Animal Care Research Compliance Office at Stanford University, adhering to the National Institutes of Health’s guidelines for the Care and Use of Laboratory Animals. The animal protocol number is 18846.

### Plasmids

Lentiviral plasmids FSW–human Synapsin 1 promoter (hSyn)–nuclear localization signal (NLS)–enhanced green fluorescent protein (EGFP)–ΔCre and FSW-hSyn-NLS-EGFP-Cre were described previously (*51*). For rescue experiments, WT BAI3 (NM_175642.4), BAI3 L856A, BAI3 L859A, BAI3 L862A, BAI3 SS− (lack of 1064 to 1096 amino acids), BAI3 T4L (replacement of 1055 to 1097 amino acids with T4L), BAI3 ΔEBD (deletion of 1411 to 1446 amino acids), and BAI3 R1433A cDNAs were inserted into the same FSW-hSyn vector by PCR or overlap extension PCR (*52*, *53*), with the original signal peptide replaced by a preprotrypsin leader signal peptide (MSALLILALVGAAVA). A Flag tag was inserted after the signal peptide, and a hemagglutinin (HA) tag was inserted before “PLDVQEGDFQTEV” in the C terminus. For BAI3 ΔECD (deletion of 1 to 856 amino acids, tethered agonist sequence remained) rescue construct, the original signal peptide was replaced by a HA signal peptide, and only an HA tag was inserted before PLDVQEGDFQTEV in the C terminus. For BAI3 DHBD/DGAIN rescue construct, the original signal peptide was replaced by a preprotrypsin leader signal peptide, followed by a Flag tag insertion; amino acids 509 to 868 were deleted, followed by the (GGGGS)_X6_ linker; and an HA tag was inserted before PLDVQEGDFQTEV in the C terminus. Lentiviral helper plasmids [pRSV-REV, pMDLg/pRRE, and vesicular stomatitis virus G protein (VSV-G)] were the same as previously used (*25*). pCAG-tdTomato was a gift from A. Bordey (Addgene). For reporter gene assays, the pGL4.30-NFAT-RE/minP/luc2 reporter plasmid and pRL-TK plasmid were gifted by S. C. Blacklow (*43*). WT or mutant BAI3 cDNAs (except BAI3 ΔECD) were inserted to pCAG vector, with the original signal peptide replaced by a preprotrypsin leader signal peptide and followed by a Flag tag. BAI3 ΔECD was inserted to pCAG vector, with the original signal peptide replaced by a preprotrypsin leader signal peptide without Flag tag insertion. pT2Aneo-tdTomato-CAAX was a gift from M. Matsuda (Addgene, plasmid no. 170284).

### Reporter gene assays

The pGL4.30-NFAT-RE/minP/luc2 reporter plasmid and pRL-TK plasmid were gifted by S. Blacklow laboratory and described in (*43*). The assays were performed similarly as described in the paper (*43*). HEK293 cells were plated into 96-well plate, each well was transfected with 50 ng of pGL4.30-NFAT-RE/minP/luc2 plasmid, 1 ng of pRL-TK plasmid, and 50 ng of pCAG-BAI3 plasmid. Fresh complete media (100 nl) were changed to the wells 6 hours posttransfection. Cells were incubated for overnight, starved in fetal bovine serum (FBS)–free Dulbecco’s modified Eagle’s medium for 6 hours. Cells were lysed using Dual Luciferase reporter kit (Promega), and luminance signals were recorded using a BMG LABTECH plate reader.

### Antibodies

For confocal microscopy imaging experiments, these antibodies were used at indicated dilution: HA (mouse; Covance, MMS101R; 1:1000); MAP2 (chicken; Encor, no. CPCA-MAP2; 1:1000); Flag (rabbit; Sigma-Aldrich, F7425; 1:500); Homer1 (rabbit; MilliporeSigma, ABN37; 1:500); vGlut1 (guinea pig; MilliporeSigma, AB5905; 1:1000); Goat anti-Mouse immunoglobulin G (IgG; H+L) Highly Cross-Adsorbed Secondary Antibody, Alexa Fluor 647 (Thermo Fisher Scientific, A-21236; 1:1500); Goat anti-Chicken IgY (H+L) Secondary Antibody, Alexa Fluor 488 (Thermo Fisher Scientific, A-11039; 1:1500); Goat anti-Rabbit IgG (H+L) Cross-Adsorbed Secondary Antibody, Alexa Fluor 546 (Thermo Fisher Scientific, A-11010; 1:1500); Goat anti-Guinea Pig IgG (H+L) Secondary Antibody, Alexa Fluor 546 (Thermo Fisher Scientific, A-11074; 1:1500); and Goat anti-Rabbit IgG (H+L) Highly Cross-Adsorbed Secondary Antibody, Alexa Fluor 647 (Thermo Fisher Scientific, A-21245; 1:1500).

For STED microscopy imaging experiments, these antibodies were used at indicated dilution: vGlut1 (rabbit; Yenzyme, 6089; 1:500); Homer1 (guinea pig, Synaptic Systems, 1:500); HA (mouse; Covance, MMS101R; 1:1,000); Flag (rabbit; Sigma-Aldrich, F7425; 1:500); Homer1 (mouse; Synaptic Systems, 160011; 1:500); vGlut1 (guinea pig; Synaptic Systems, 135304; 1:500); abberior STAR ORANGE, goat anti-rabbit IgG (abberior, STORANGE-1002, 1:500); abberior STAR RED, goat anti-guinea pig IgG (abberior, STRED-1006, 1:500); abberior STAR 460L, goat anti-mouse IgG (abberior, ST460L-1001, 1:500); abberior STAR 460L, goat anti-rabbit IgG (abberior, ST460L-1002, 1:500); abberior STAR RED, goat anti-mouse IgG (abberior, STRED-1001, 1:500); and STAR ORANGE, goat anti-guinea pig IgG (abberior, STORANGE-1006, 1:500).

### Primary hippocampal culture of mixed neurons and glia

As previously described (*46*), at DIV0, hippocampi were isolated from postnatal day 0 mice and subjected to papain digestion (Worthington) for 20 min at 37°C. The digested tissue was then passed through a 70-μm cell strainer and placed on Matrigel (Corning)–coated coverslips in 24-well plates. The plating medium consisted of 5% FBS (Atlanta), B27 supplement (Gibco), 0.4% glucose (MilliporeSigma), and 2 mM glutamine (Gibco) dissolved in minimum essential medium (MEM; Gibco). At DIV1, the culture medium was replaced with a growth medium composed of 5% FBS (Atlanta), B27 (Gibco), and 2 mM glutamine (Gibco) in Neurobasal-A (Gibco). At DIV 4, half of the medium was substituted with growth medium containing 8 μM Ara-C (MilliporeSigma), achieving a final concentration of 4 μM (MilliporeSigma). Cultures were evaluated at DIV14.

### Lentivirus production

Lentiviruses were generated by cotransfecting Lenti-X 293 T cells with a lentiviral plasmid (12 μg per T75 flask) along with three helper plasmids: pRSV-REV (4 μg), pMDLg/pRRE (8 μg), and VSV-G (6 μg) per T75 flask. After 48 hours, the lentivirus-containing medium was collected, concentrated using PEG-it Virus Precipitation Solution (System Biosciences), resuspended in 100 μl of MEM, aliquoted, and stored at −80°C. ΔCre and Cre lentiviruses (1 to 2 μl) were first applied at DIV4 to achieve efficient gene deletion, followed by the addition of 3 μl of BAI3 rescue lentiviruses at DIV6. For lentiviral transduction efficiency in neurons and glia, please see fig. S4 of the previously published paper (*20*).

### Expression levels of WT and mutant BAI3 proteins

Primary hippocampal cultures at DIV 14 were washed once with prewarmed phosphate-buffered saline (PBS) and fixed in a solution of 4% paraformaldehyde (PFA) and 4% sucrose in PBS for 20 min at 4°C. After three washes with PBS, the cultures were blocked with a buffer containing 2.5% goat serum (MilliporeSigma) and 2.5% bovine serum albumin (BSA) in PBS (without permeabilization). The Flag antibody was applied in the same blocking buffer and incubated overnight at 4°C. Following three PBS washes, the secondary antibody was applied in the same blocking buffer for 1 hour at room temperature. Subsequently, the cultures were washed three times with PBS and refixed in 4% PFA and 4% sucrose in PBS for 20 min at 4°C. After three more PBS washes, the cultures were blocked in a new buffer containing 2.5% goat serum (MilliporeSigma), 2.5% BSA, and 0.2% Triton X-100 (with permeabilization) in PBS for 1 hour at room temperature. The HA and MAP2 antibodies were then applied in the new blocking buffer and incubated overnight at 4°C. Following another three PBS washes, the cultures were incubated with the secondary antibodies in the same new blocking buffer for 1 hour at room temperature. After a final set of three PBS washes, the coverslips were mounted on microscope slides using 4′,6-diamidino-2-phenylindole (DAPI) Fluoromount-G (SouthernBiotech, 0100-20). Imaging was conducted using a Nikon A1RSi confocal microscope with a 60× objective lens. Fiji software was used for image analysis, with the intensity ratio of HA/MAP2 or Flag/MAP2 serving as a metric for expression levels.

### Axonal and dendritic arborization, and soma area analysis

As outlined previously (*25*, *54*), hippocampal cultures were sparsely transfected with CAG-tdTomato using calcium phosphate transfection at DIV9. For axon tracing, 0.5 μg of DNA per well was used in 24-well plates, whereas, for dendrite arborization and soma area analysis, 1.0 μg of DNA per well was applied. At DIV14, cells were rinsed once with PBS and then fixed with a solution containing 4% PFA, 4% sucrose, and PBS for 20 min at 4°C. After an additional three washes with PBS, coverslips were mounted onto microscope slides using DAPI Fluoromount-G (SouthernBiotech, 0100-20). Images were captured using a Nikon A1RSi confocal microscope, with 10× objective lens for axon tracing and 60× objective lens for dendrite morphology and soma area. Soma area was analyzed in Fiji software. Axonal and dendritic arborizations were analyzed using the Simple Neurite Tracer plugin in Fiji software (*55*, *56*).

### Spine morphology

Hippocampal cultures in a 24-well plate were sparsely transfected with 1 μg of pT2Aneo-tdTomato-CAAX per well using calcium phosphate transfection DIV 9. At DIV 14, the cells were rinsed once with PBS and subsequently fixed in a solution containing 4% PFA, 4% sucrose, and PBS for 20 min at 4°C. Following fixation, the cells were washed three times with PBS, and coverslips were mounted onto microscope slides using DAPI Fluoromount-G (SouthernBiotech, 0100-20). Imaging was performed using a Nikon A1RSi confocal microscope equipped with a 100× objective lens. The analysis of spine morphology was conducted automatically using Imaris software (*57*).

### Synapse density analyses

For the vGlut1/Homer1/MAP2 immunocytochemistry experiments, at DIV14, primary hippocampal cultures were washed with prewarmed PBS, followed by fixation in a 4% PFA, 4% sucrose, and PBS solution for 20 min at 4°C. After fixation, the cultures underwent three PBS washes and were blocked in a buffer consisting of 2.5% goat serum (MilliporeSigma), 2.5% BSA, and 0.2% Triton X-100 (for permeabilization) in PBS for 1 hour at room temperature. The primary antibodies for vGlut1, Homer1, and MAP2 were applied in the blocking buffer and incubated overnight at 4°C. Following this, the cultures were washed three times with PBS and incubated with fluorescence-labeled secondary antibodies in the same blocking buffer for 1 hour at room temperature. The cultures were washed three more times with PBS, and coverslips were mounted on microscope slides using DAPI Fluoromount-G (SouthernBiotech, 0100-20).

Images were taken on a Nikon A1RSi confocal microscope with a 60× objective lens. Synaptic puncta quantification was carried out using Nikon NIS-Elements (Nikon) as previously described (*58*). For the same batch of samples, the average background intensities for each channel were calculated and subtracted from each image. A consistent binary mask was applied across all images within the same batch. For each neuron, three regions of interest (ROIs) were delineated along three separate primary dendrites. The soma was centered in the image, and each ROI covered the entire primary dendrite from the edge of the soma to the image edge, about 100 μm in length. Synapse density was calculated as the number of colocalized vGlut1 and Homer1 divided by the length of the dendritic segment. The synapse density for each neuron was determined by averaging the synapse densities from the three ROIs.

### STED microscopy imaging

For HA/vGlut1/Homer1 immunocytochemistry experiments, at DIV14, primary hippocampal cultures were washed with PBS and fixed with 4% PFA and 4% sucrose in PBS for 20 min at 4°C. After fixation, cells were washed three times with PBS and blocked for 1 hour at room temperature with PBS containing 5% goat serum (MilliporeSigma) and 0.2% Triton X-100 (permeabilization). Primary antibodies against HA, vGlut1, and Homer1 were applied in the same blocking buffer and incubated overnight at 4°C. Following primary antibody incubation, cells were washed three times with PBS and treated with fluorescence-labeled secondary antibodies for 1 hour at room temperature. After three additional PBS washes, the coverslips were mounted on slides using ProLong Gold Antifade Mountant (Thermo Fisher Scientific, P36930).

For Flag/vGlut1/Homer1 immunocytochemistry experiments, at DIV14, primary hippocampal cultures were washed with PBS and fixed with 4% PFA and 4% sucrose in PBS for 20 min at 4°C. After fixation, cells were washed three times with PBS and blocked for 1 hour at room temperature with PBS containing 5% goat serum (MilliporeSigma). Primary antibody for Flag was applied in the same blocking buffer and incubated overnight at 4°C. Cells were washed three times with PBS and treated with secondary antibodies for 1 hour at room temperature. After three PBS washes, the cells were refixed in 4% PFA and 4% sucrose in PBS for 20 min at 4°C. After three more PBS washes, the cultures were blocked in a new buffer containing 5% goat serum (MilliporeSigma) and 0.2% Triton X-100 (for permeabilization) in PBS for 1 hour at room temperature. Primary antibodies against vGlut1 and Homer1 were applied in the new blocking buffer and incubated overnight at 4°C. Following another three PBS washes, the cultures were incubated with the secondary antibody in the same new blocking buffer for 1 hour at room temperature. After a final set of three PBS washes, the coverslips were mounted on microscope slides using ProLong Gold Antifade Mountant (Thermo Fisher Scientific, P36930).

All images were captured using a Nikon Ti2-E microscope stand equipped with a STEDYCON confocal and STED module from Abberior Instruments Inc. Image analysis was performed using Huygens Software and Nikon NIS-Elements, as described previously (*58*). A consistent binary mask was applied to all images within each batch. For each neuron, three polygonal ROIs were drawn. A “synapse” was characterized as “vGlut1 having (at least one pixel overlap with another mask) Homer1 or Homer1 having vGlut1.” Synaptic BAI3 was defined as the proportion of BAI3 having a synapse. For each neuron, the average value from the three ROIs was calculated and used.

### Electrophysiology

Traditional whole-cell patch-clamp recordings were performed from cultured hippocampal neurons plated on coverslips, which were placed in a recording chamber mounted on a fixed-stage inverted phase-contrast microscope (Olympus). Patch electrodes (3 to 5 MΩ) were made from borosilicate glass capillary tubes (Warner Instruments) using a PC-10 pipette puller (Narishige). Whole-cell capacitance and series resistances were recorded and compensated to >80%, and, in addition, series resistances were less than two times the tip resistance.

All recordings were conducted under voltage-clamp mode with a pipette solution containing: 135 mM CsCl, 10 mM Hepes, 10 mM EGTA, 2 mM Mg-ATP, 2 mM Na2GTP, and 5 mM QX-314 (pH 7.35; adjusted with CsOH). mEPSCs were pharmacologically isolated with picrotoxin (50 μM) and tetrodotoxin (1 μM) in the normal Tyrode’s bath solution containing: 129 mM NaCl, 5 mM KCl, 2 mM CaCl_2_, 1 mM MgCl_2_, 0.01 mM glycine, 30 mM d-glucose, and 25 mM Hepes, and recorded at a −70-mV holding potential.

For evoked EPSC recording, the presynaptic action potential for evoked synaptic responses was triggered by 0.5-ms current (40 to 90 μA) injections through a local extracellular electrode (FHC concentric bipolar electrode, catalog no. CBAEC75) placed ~100 μm from the soma of the recorded neurons. The frequency, duration, and magnitude of the extracellular stimulus were controlled with a Pulse Stimulator (A-M Systems Inc.) synchronized with the Clampex 10.2 data acquisition software (Molecular Devices). To determine paired-pulse ratios of evoked synaptic responses, two sequential action potentials (APs) were elicited with a given time interval. Each paired stimulation interval (20, 50, 100, 500, and 1000 ms) was repeated three times with an intertrial interval of 30 s.

All data were filtered during acquisition with a low pass filter set at 2 kHz using pClamp 10 (Molecular Devices). Data analysis was performed offline with Clampfit 10.2 (Molecular Devices)

### Quantifications, statistical analyses, and list of data shared among figures

Statistical analyses were performed using GraphPad Prism 10 software. Comparisons between two groups were conducted using unpaired two-tailed Student’s *t* tests. For comparisons involving more than two groups, one-way analysis of variance (ANOVA) followed by Dunnett’s multiple comparisons test or nested one-way ANOVA followed by Dunnett’s multiple comparisons test were used as noted. Two-way ANOVA was used for multifactorial analysis. The Kolmogorov-Smirnov test was used for analyzing cumulative distributions in electrophysiological recordings. For all statistical analyses, the significance levels were set as follows: *P < 0.05, **P < 0.01, ***P < 0.001, and ****P < 0.0001.

All raw data for this paper are publicly available at the Stanford Data Repository (https://purl.stanford.edu/bj843yr3863). Please note that, for several figures, data were derived from experiments where multiple questions were addressed simultaneously. As a result, data from the same control condition were used across multiple figures whenever experiments were done at the same time. Moreover, some experiments addressing a distinct set of questions about the mechanism of BAI3 function that are reported in an earlier manuscript (*20*) also partly used the same control conditions. Specifically, (i) all reporter gene assays in this paper (Figs. 1C, 5B, and 8B) share the same WT control group data; (ii) “Cre” and “Cre + BAI3-WT” group data in figs. S1C and S5B are identical; (iii) “ΔCre,” Cre, and Cre + BAI3-WT group data in Fig. 2 (B and C) are identical to those in Fig. 5 (D and E); (iv) part of the ΔCre and Cre group data in Figs. 2 (B and C) and 5 (D and E) are identical to those in figure 4g of the second paper; (v) Cre + BAI3-WT group data in Figs. 2 (B and C) and 5 (D and E) are identical to those in figure 4g of the second paper; (vi) part of the ΔCre and Cre group data in Fig. 5 (G and H) and fig. S5C are identical to those in Fig. 2 (E and F) and fig. S1D of this paper and figure 4e and supplementary figure 7c of the second paper; (vii) Cre + BAI3-WT group data in Fig. 2 (E and F) and fig. S1D are identical to those in Fig. 5 (G and H) and fig. S5C of this paper and figure 4e and supplementary figure 7c of the second paper; (viii) part of the ΔCre, Cre, and Cre + BAI3-WT group data in Fig. 6B are identical to those in Fig. 3B of this paper and figure 5 of the second paper; (ix) Cre + BAI3-WT group data in Fig. 4E are identical to those in Fig. 7B; (x) part of Cre + BAI3-WT group data in Figs. 4E and 7B of this paper are identical to those in figure 7b of the second paper; (xi) ΔCre and Cre group data in Fig. 4 (A to C) are identical to those in fig. S7 (C to E); and (xii) ΔCre and Cre group data in fig. S4 (B1 and B2) are identical to those in fig. S7 (F and G).
