# Supplementary material for: Tethered agonist– and GAIN domain–independent signaling of an adhesion GPCR
Source: Sci Adv. 2026 Jul 1;12(27):eadu3822. doi: 10.1126/sciadv.adu3822 (PMC13322242; doi:10.1126/sciadv.adu3822)
Supplement: Supplementary file 1 — Figs. S1 to S8 [file sciadv.adu3822_sm.pdf]

Science Advances
AAAS

Supplementary Materials for

Tethered agonist– and GAIN domain–independent signaling of an adhesion GPCR

Jie Wang *et al.*

Corresponding author: Jie Wang, jvwang@ust.hk; Yi Miao, yimiao@ust.hk; Thomas C. Südhof, tcs1@stanford.edu

Sci. Adv. **12**, eadu3822 (2026)
DOI: 10.1126/sciadv.adu3822

This PDF file includes:

Figs. S1 to S8

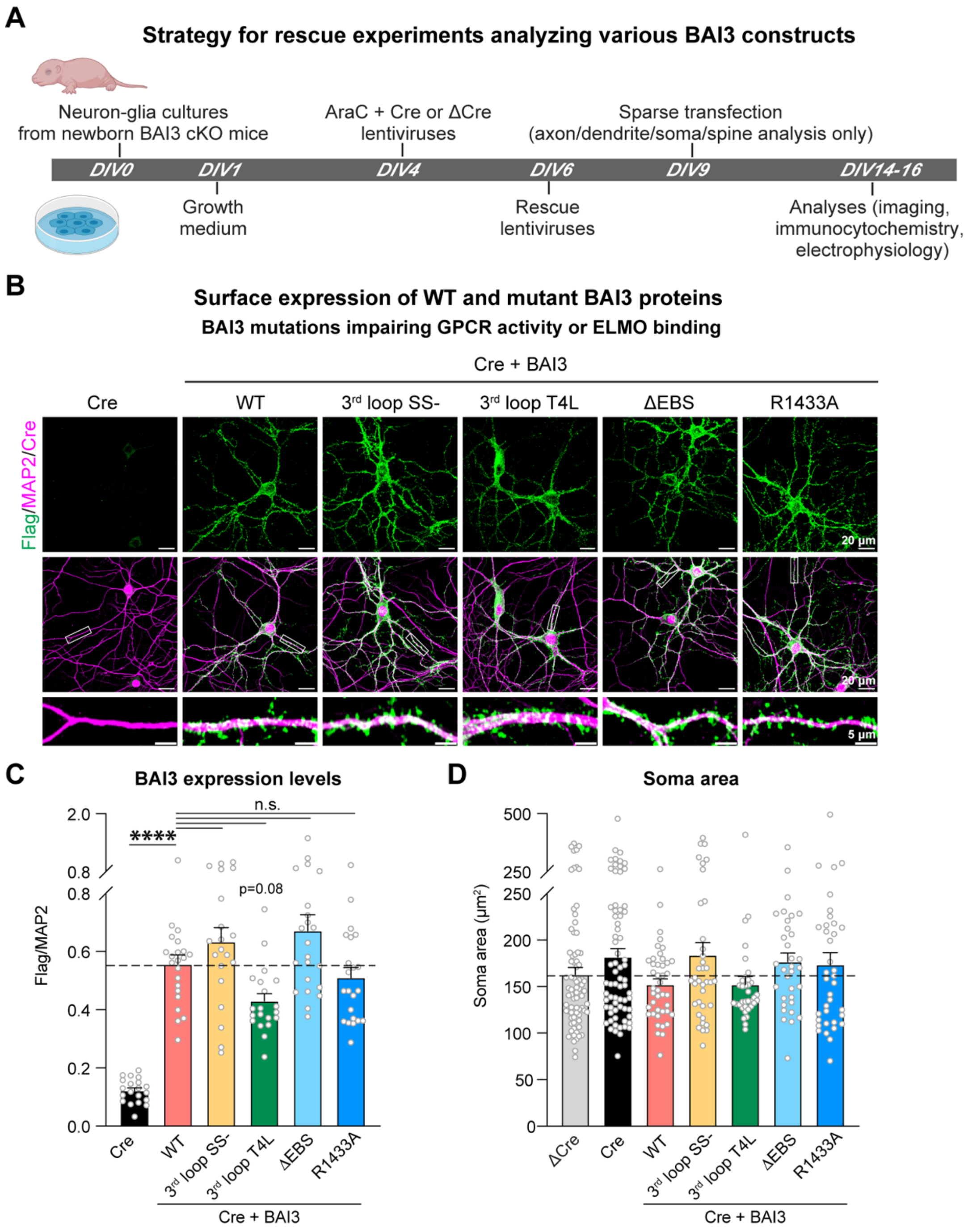

Fig. S1. Strategy for experiments analyzing BAI3 signaling mechanisms (A), surface expression of wild-type (WT) and mutant BAI3 proteins used for Fig. 1-4 in hippocampal neuron-glia cultures (B & C), and lack of effect of BAI3 deletion and rescue interventions on soma size of hippocampal neurons (D)

(A) Detailed experimental timeline for the experiments investigating the molecular mechanisms underlying the function of BAI3 in restricting axonal and dendritic arborization and in supporting

synapse formation and synaptic transmission. Created in BioRender. Miao, Y. (2026) https://BioRender.com/6ryzwic.

(**B**) Representative images of hippocampal neurons from mixed neuron-glia cultures, showing staining for MAP2 (magenta) and Flag-tagged BAI3 (green). Cultures were derived from BAI3 cKO mice, infected with Cre (magenta) lentivirus at DIV4 and BAI3-expressing lentiviruses at DIV6 using the constructs described in Fig. 1-4, followed by immunocytochemistry at DIV14 (top panel: separate Flag channel overview; middle panel: full-neuron overview; bottom panel: zoomed-in area for the white rectangle in the overview).

(**C**) Summary graphs of the surface expression levels of Flag-tagged wild-type and mutant BAI3 in BAI3-deficient neurons. Flag/MAP2 signal ratio was calculated. N = 20/3, 22/3, 20/3, 20/3, 22/3, and 22/3 (cells/experiments) from left to right.

(**D**) Summary graphs of soma area for experiments in Fig. 2D. N (cells/experiments) = 79/11, 70/11, 42/7, 37/6, 35/4, 33/7, and 37/6 from left to right.

Data in (C) and (D) are mean values ± SEM. Statistical significance was determined using one-way ANOVA followed by Dunnett's multiple comparison tests comparing all conditions to WT (C) or ΔCre (D) (**** $p<0.0001$).

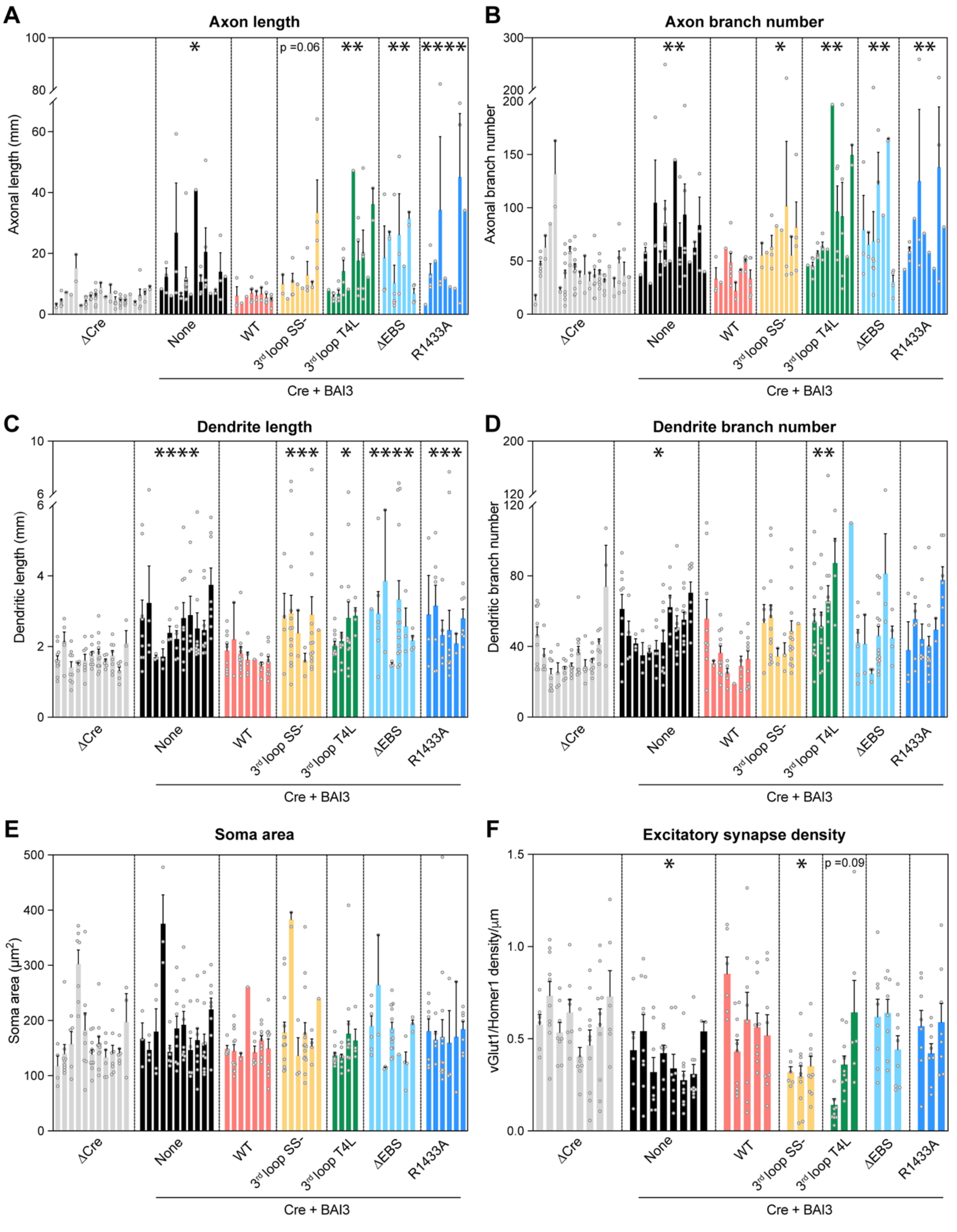

Fig. S2. Nested one-way ANOVA analyses of axonal growth, dendritic arborization, soma area, and excitatory synapse density for BAI3 wild-type (WT) and mutants impairing GPCR activity and ELMO binding in order to compare means across groups and to evaluate the

variation not only between main groups but also between subgroups as a more rigorous statistical assessment

(**A**-**D**) Nested one-way ANOVA analyses of axon length (A), axon branch number (B), dendrite length (C), and dendrite branch number (D) for Fig. 2.

(**E**) Nested one-way ANOVA analyses of the soma area for Fig. S1D.

(**F**) Nested one-way ANOVA analyses of excitatory synapse density for Fig. 3B.

Data are means ± SEM. Each column represents a different experiment, and individual dots represent individual cells. Statistical significance was determined using nested one-way ANOVA followed by Dunnett's multiple comparison tests comparing all conditions to ΔCre controls (* $p<0.05$, ** $p<0.01$, *** $p<0.001$, **** $p<0.0001$).

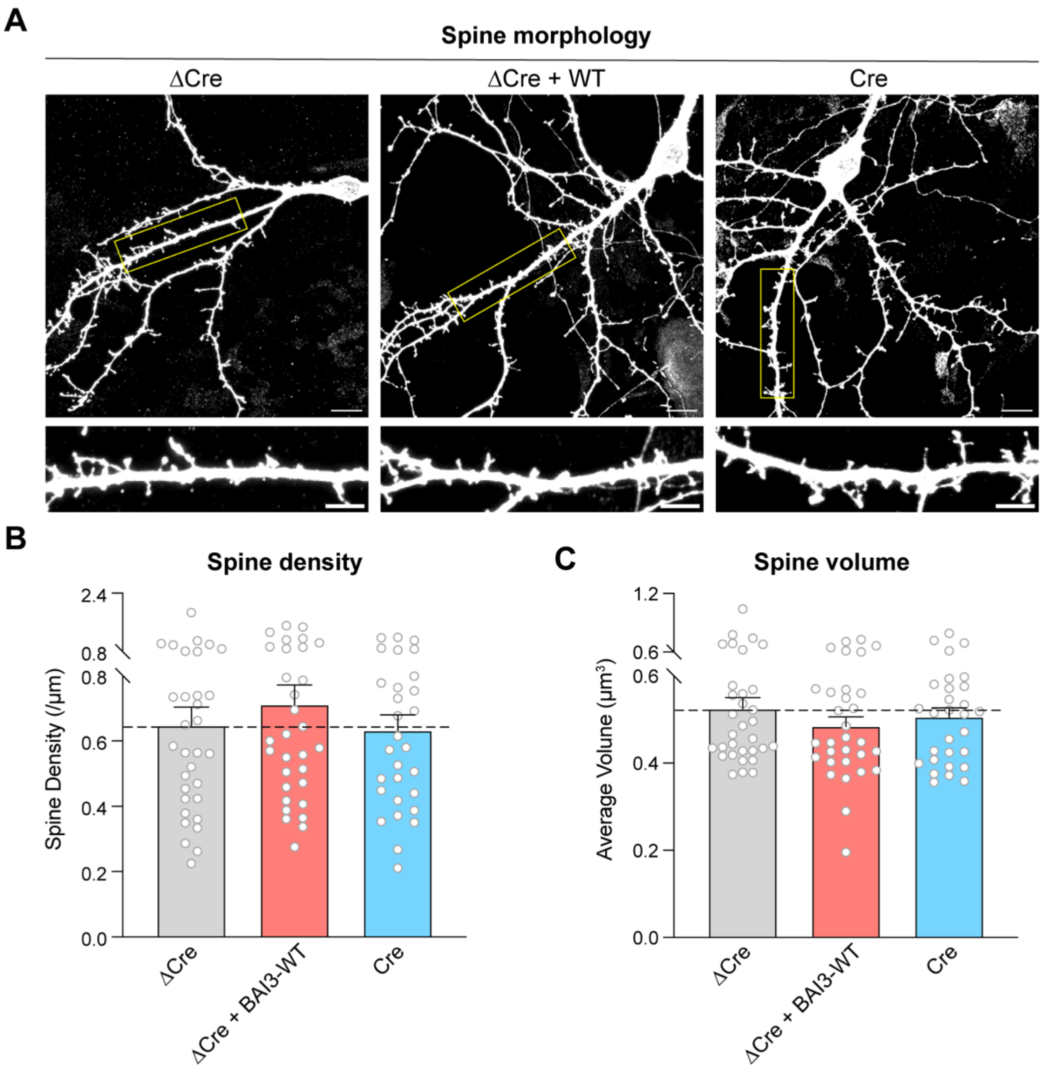

Fig. S3. BAI3 deletion and overexpression have no effect on spine density and volume in hippocampal culture neuron

(**A**) Representative images of cultured hippocampal neurons used to assess the impact of BAI3 deletion or overexpression on spine morphology. Mixed neuron-glia cultures derived from newborn BAI3 conditional KO mice were infected at DIV4 with lentiviruses expressing ΔCre-EGFP (control) or Cre-EGFP fusion proteins, and subsequently infected at DIV6 with lentivirus expressing wild-type (WT) BAI3. To analyze spines, neurons were sparsely transfected with tdTomato-CAAX expression plasmids at DIV9 to label individual neurons, and analysis was performed at DIV14. The lower images are magnified views of the yellow boxes in the upper images. Scale bars represent 10 μm (upper) and 5 μm (lower).

(**B & C**) Quantification of spine density (B) and spine volume (C) from the experiments shown in (A). Data are presented as means ± SEM. N = 32/3, 31/3, and 29/3 from left to right. Statistical significance was assessed using one-way ANOVA followed by Dunnett's multiple comparisons test, comparing all conditions to ΔCre controls.

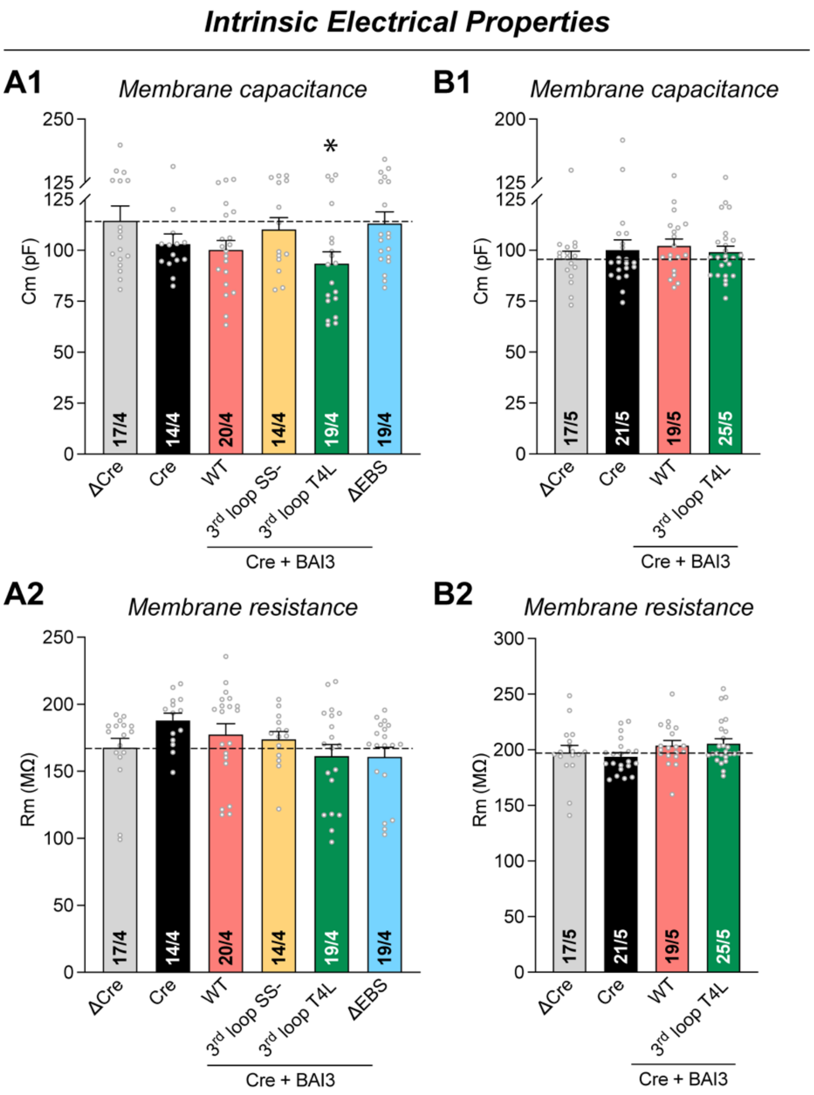

Fig. S4. BAI3 deletion and rescue interventions have no effect on neuronal intrinsic electrical properties

(A - B) Quantifications of membrane capacitance (A1, B1) and membrane resistance (A2, B2) during the electrophysiological recordings described in Fig. 3C-3E (A1 & A2) and Fig. 4A-4C (B1 & B2). Results are presented as mean values ± SEM, with cell/experiment numbers shown in the bars. Statistical significance was determined using one-way ANOVA followed by Dunnett's multiple comparison tests comparing all conditions to ΔCre (* $p<0.05$).

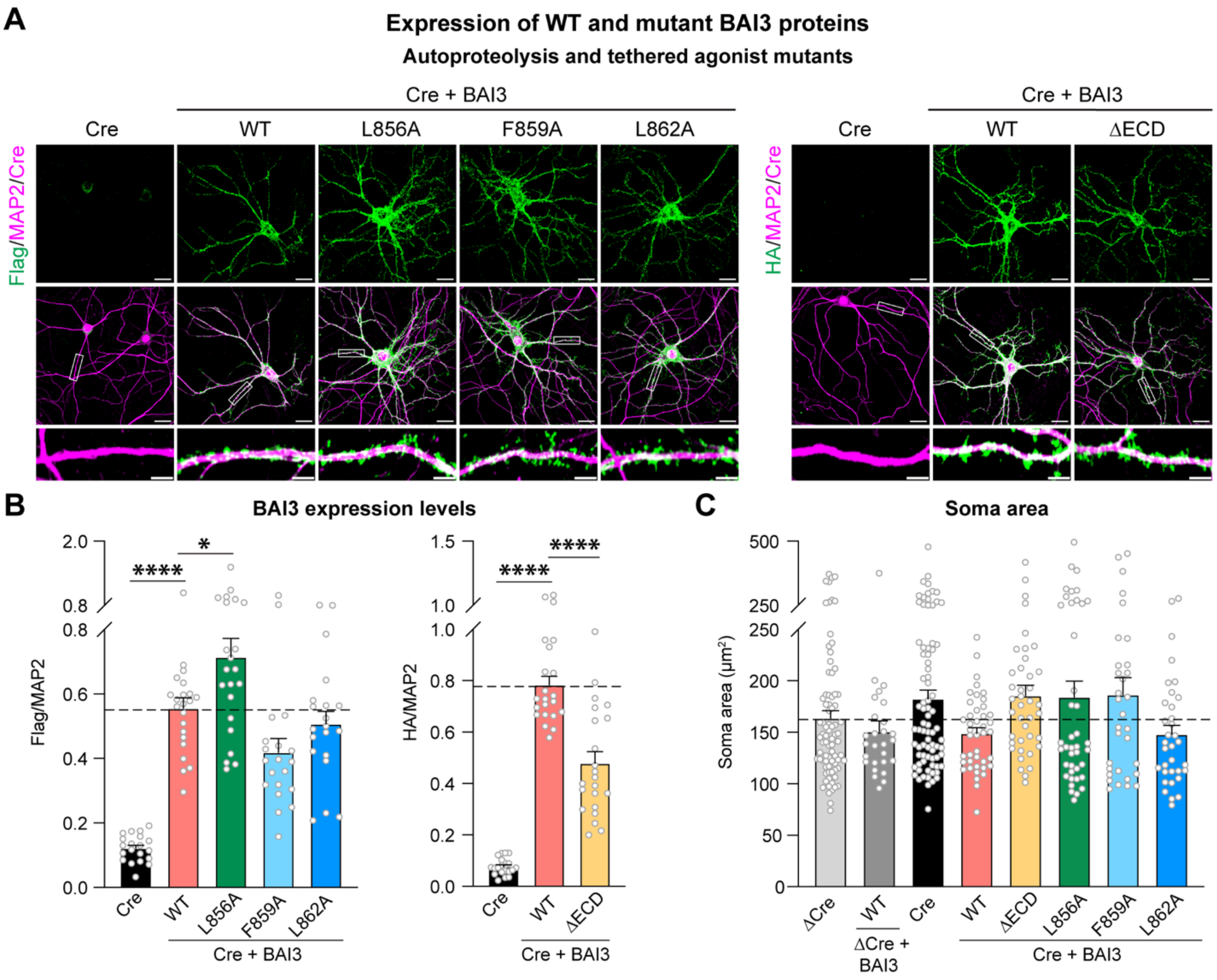

Fig. S5. Expression levels of wild-type (WT) and mutant BAI3 proteins used for Fig. 5-7 in hippocampal neuron-glia cultures (A and B), and demonstration that the various BAI3 manipulations used in these figures have no effect on soma size of hippocampal neurons (C)

(**A**) Representative images of hippocampal neurons from mixed neuron-glia cultures, showing staining for MAP2 (magenta) and Flag (left)- or HA (right)-tagged BAI3 (green) (top panel: separate Flag or HA channel overview; middle panel: full-neuron overview; bottom panel: zoomed-in area for the white rectangle in the overview). Experiments were performed as described in Fig. S1B. Scale bars represent 20 μm (upper and middle) and 5 μm (lower).

(**B**) Summary graphs of the expression levels of Flag- or HA-tagged wild-type and mutant BAI3 in BAI3-deficient neurons. Flag/MAP2 (left) or HA/MAP2 (right) signal ratio was calculated. Please note that the Cre and Cre + BAI3-WT groups are the same as those in Fig. S1C (see method details). N = 20/3, 22/3, 22/3, 20/3, and 20/3 (cells/experiments) for Flag/MAP2, and 20/3, 22/3, and 22/3 for HA/Flag.

(**C**) Summary graphs of soma area for experiments in Fig. 5F. Please note that the ΔCre, Cre, and Cre + BAI3-WT groups have overlapping or same samples as those in Fig. S1D (see method

details). N (cells/experiments) = 82/12, 27/6, 79/12, 42/7, 40/4, 41/9, 32/7, and 34/6 from left to right.

Data in (B) and (C) are mean values ± SEM. Statistical significance was determined using one-way ANOVA followed by Dunnett's multiple comparison tests comparing all conditions to WT (B) or ΔCre (C) (* $p<0.05$, **** $p<0.0001$).

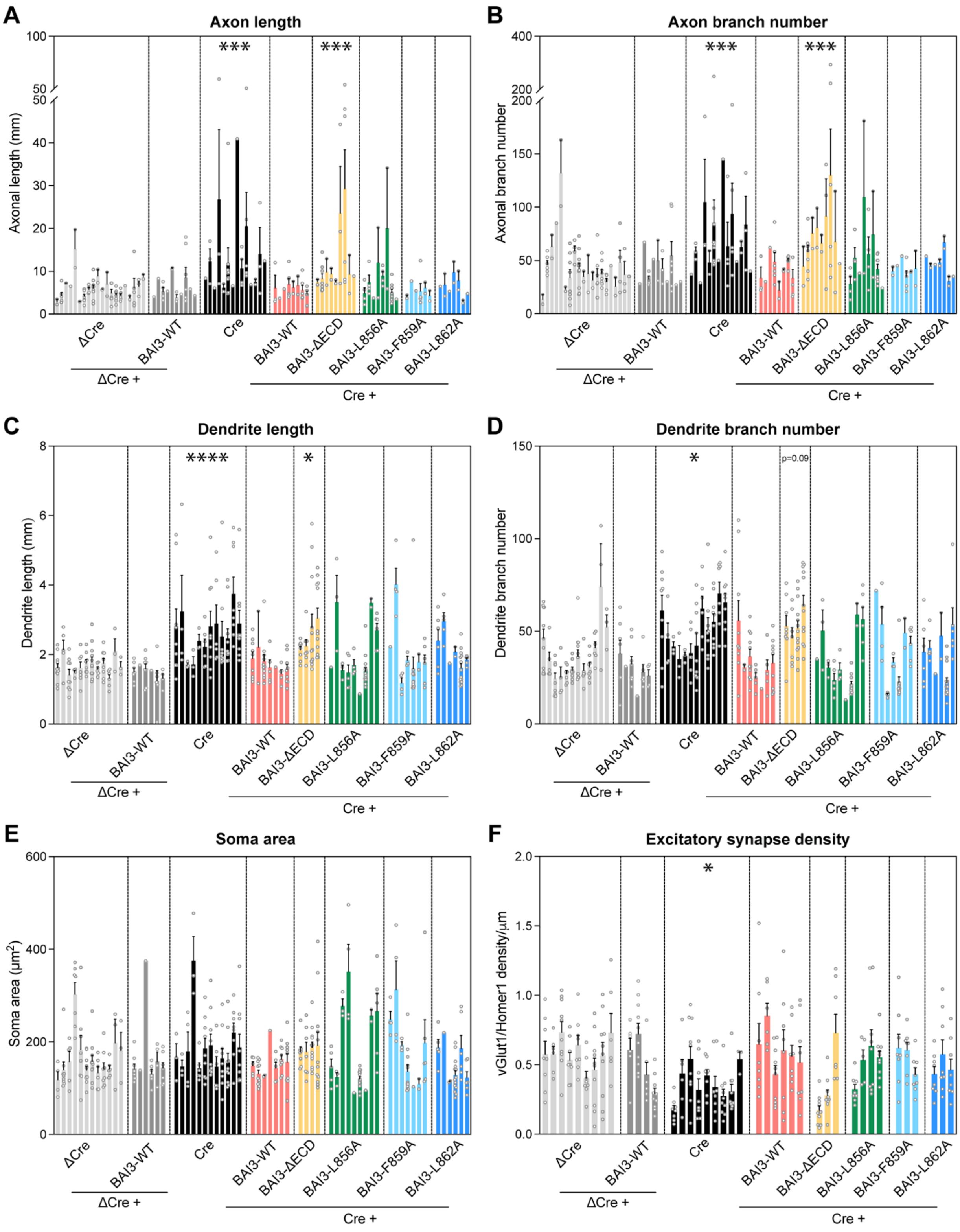

Fig. S6. Nested one-way ANOVA analyses of axonal growth, dendritic arborization, soma area, and excitatory synapse density for BAI3 wild-type (WT) and mutants disrupting autoproteolysis and tethered agonist activity to compare means across groups and to

evaluate the variation not only between main groups but also between subgroups as a more rigorous statistical assessment

(A-D) Nested one-way ANOVA analyses of axon length (A), axon branch number (B), dendrite length (C), and dendrite branch number (D) for Fig. 5.

(**E**) Nested one-way ANOVA analyses of soma area for Fig. S5C.

(**F**) Nested one-way ANOVA analyses of excitatory synapse density for Fig. 6B.

Data are means ± SEM. Each column represents a different experiment, and individual dots represent individual cells. Statistical significance was determined using nested one-way ANOVA followed by Dunnett's multiple comparison tests comparing all conditions to ΔCre controls (* $p<0.05$, *** $p<0.001$, **** $p<0.0001$).

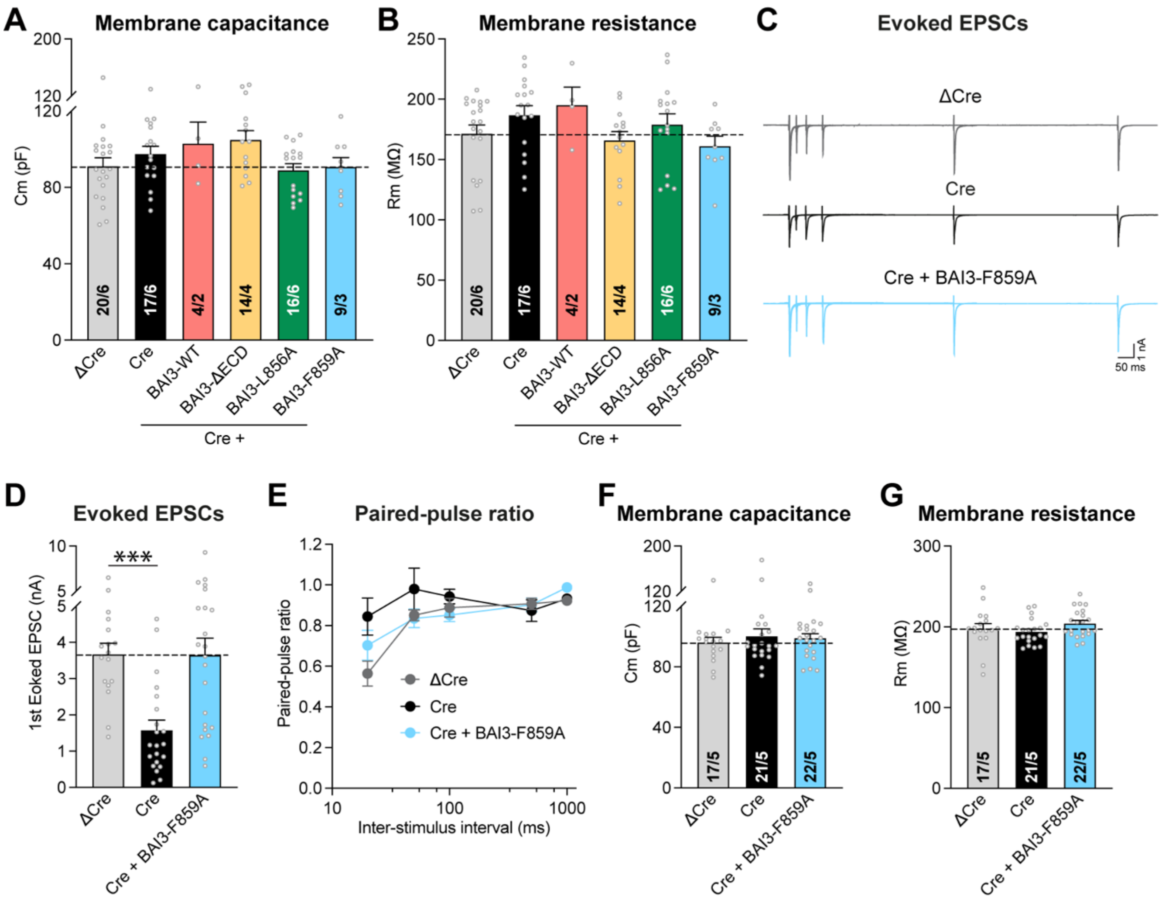

Fig. S7. BAI3 deletion and rescue interventions used in Fig. 5-7 have no effect on neuronal intrinsic electrical properties (A and B), and mutating the tethered agonist of BAI3 has no effect on BAI3's ability to sustain wild-type evoked EPSC amplitudes (C-E) and also does not alter the intrinsic electrical properties of neurons (F and G)

(A & B) Quantifications of membrane capacitance (A) and membrane resistance (B) for the electrophysiological recordings described in Fig. 6C-6E.

(**C**) Representative traces of evoked EPSCs monitored in hippocampal neurons expressing the indicated lentiviruses. EPSCs were evoked by closely spaced pairs of action potentials elicited by extracellular stimulation with a concentric electrode. Note that the data for the Cre and ΔCre (control) conditions in panels C-G are the same as those used for the experiments described in Fig. 4A-4C and S4B1-S4B2 since the two sets of experiments were performed at the same time.

(**D**) Summary graph of the amplitude of the first evoked EPSC during paired-pulse stimulation for the experiments shown in (C). N = 17/5, 21/5, and 22/5 (cells/experiments) from left to right.

(**E**) Summary plot of the paired-pulse ratios (PPRs) of evoked EPSC amplitudes as a function of the inter-stimulus interval for the experiments depicted in (C).

(F & G) Quantifications of membrane capacitance (F) and membrane resistance (G) for the electrophysiological recordings described in (C).

All numerical data are means ± SEM. The numbers of cells/experiments are indicated in the bars in A, B, F, and G. Statistical significance was determined using one-way ANOVA followed by Dunnett's multiple comparison tests in all bar graphs, or two-way ANOVA for summary plot in (E) (*** $p<0.001$).

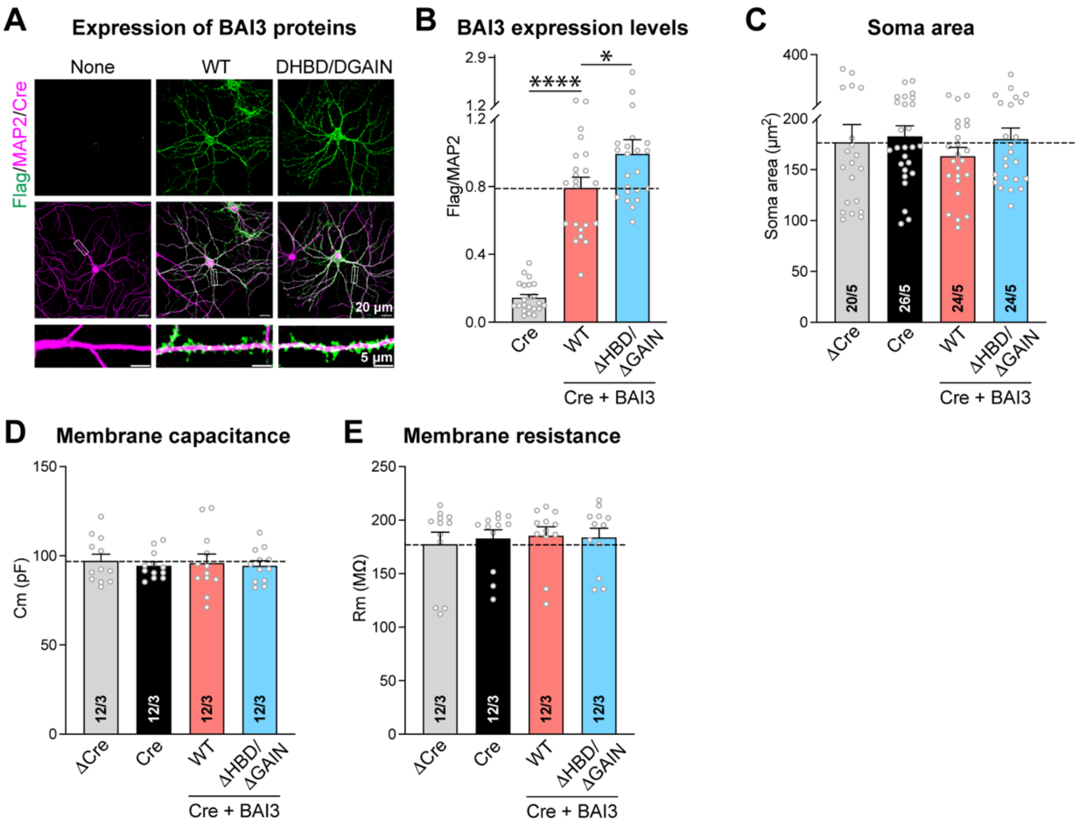

Fig. S8. Expression levels of wild-type (WT) and mutant BAI3 proteins used in Fig. 8-9 in hippocampal neuron-glia cultures (A & B), and demonstration that the manipulations described in Fig. 8-9 have no effect on soma size (C) or intrinsic electrical properties of hippocampal neurons (D & E)

(**A**) Representative images of hippocampal neurons from mixed neuron-glia cultures, showing staining for MAP2 (magenta) and surface Flag-tagged BAI3 (green) (top panel: separate Flag channel overview; middle panel: full-neuron overview; bottom panel: zoomed-in area for the white rectangle in the overview). Cultures were derived from BAI3 cKO mice, infected with Cre (magenta) lentivirus at DIV4 and BAI3-expressing lentiviruses at DIV6, followed by immunocytochemistry at DIV14.

(**B**) Summary graphs of the expression levels of surface Flag-tagged wild-type and mutant BAI3 in BAI3-deficient neurons. Flag/MAP2 signal ratio was calculated. N = 22/3, 22/3, and 22/3 (cells/experiments) from left to right.

(**C**) Summary graphs of soma area for experiments in Fig. 8E.

(D & E) Quantifications of membrane capacitance (D) and membrane resistance (E) for the electrophysiological recordings described in Fig. 9C-9E.

All numerical data are means ± SEM. The numbers of cells/experiments are indicated in the bars in C, D, and E. Statistical significance was determined using one-way ANOVA followed by

Dunnett's multiple comparison tests, comparing all groups to WT (B) or ΔCre (C, D, and E) (* $p<0.05$, **** $p<0.0001$).
